# Cestode strobilation: prediction of developmental genes and pathways

**DOI:** 10.1186/s12864-020-06878-3

**Published:** 2020-07-16

**Authors:** Gabriela Prado Paludo, Claudia Elizabeth Thompson, Kendi Nishino Miyamoto, Rafael Lucas Muniz Guedes, Arnaldo Zaha, Ana Tereza Ribeiro de Vasconcelos, Martin Cancela, Henrique Bunselmeyer Ferreira

**Affiliations:** 1 Laboratório de Genômica Estrutural e Funcional, Centro de Biotecnologia (CBiot), Universidade Federal do Rio Grande do Sul (UFRGS), Porto Alegre, RS Brazil; 2 Programa de Pós-Graduação em Biologia Celular e Molecular, CBiot, UFRGS, Porto Alegre, RS Brazil; 3 Departamento de Farmacociências, Universidade Federal de Ciências Médicas de Porto Alegre, Porto Alegre, RS Brazil; 4 Laboratório Nacional de Computação Científica, Petrópolis, RJ Brazil; 5 Present address: Instituto Hermes Pardini, Vespasiano, MG Brazil

**Keywords:** Platyhelminthes, Segmentation, Development, Comparative genomics, Co-expression network

## Abstract

**Background:**

Cestoda is a class of endoparasitic worms in the flatworm phylum (Platyhelminthes). During the course of their evolution cestodes have evolved some interesting aspects, such as their increased reproductive capacity. In this sense, they have serial repetition of their reproductive organs in the adult stage, which is often associated with external segmentation in a developmental process called strobilation. However, the molecular basis of strobilation is poorly understood. To assess this issue, an evolutionary comparative study among strobilated and non-strobilated flatworm species was conducted to identify genes and proteins related to the strobilation process.

**Results:**

We compared the genomic content of 10 parasitic platyhelminth species; five from cestode species, representing strobilated parasitic platyhelminths, and five from trematode species, representing non-strobilated parasitic platyhelminths. This dataset was used to identify 1813 genes with orthologues that are present in all cestode (strobilated) species, but absent from at least one trematode (non-strobilated) species. Development-related genes, along with genes of unknown function (UF), were then selected based on their transcriptional profiles, resulting in a total of 34 genes that were differentially expressed between the larval (pre-strobilation) and adult (strobilated) stages in at least one cestode species. These 34 genes were then assumed to be strobilation related; they included 12 encoding proteins of known function, with 6 related to the Wnt, TGF-β/BMP, or G-protein coupled receptor signaling pathways; and 22 encoding UF proteins. In order to assign function to at least some of the UF genes/proteins, a global gene co-expression analysis was performed for the cestode species *Echinococcus multilocularis*. This resulted in eight UF genes/proteins being predicted as related to developmental, reproductive, vesicle transport, or signaling processes.

**Conclusions:**

Overall, the described in silico data provided evidence of the involvement of 34 genes/proteins and at least 3 developmental pathways in the cestode strobilation process. These results highlight on the molecular mechanisms and evolution of the cestode strobilation process, and point to several interesting proteins as potential developmental markers and/or targets for the development of novel antihelminthic drugs.

## Background

The phylum Platyhelminthes comprise a diverse array of species that occur in all continental masses, seas, rivers, and lakes. Platyhelminths (flatworms) are dorsoventrally flattened bilaterian acoelomates that lack an anus, possess a low level of cephalization, and are usually hermaphroditic [1]. They are divided into four main classes: Turbellaria (free-living planarians), Monogenea (mostly aquatic ectoparasites), Trematoda (flukes), and Cestoda (tapeworms) [2].

Parasitic flatworms (monogeneans, flukes, and tapeworms) form a monophyletic group known as Neodermata [3], which constitutes one of the three largest groups of metazoan parasites that infect vertebrates (the others being nematodes and arthropods). Flukes and tapeworms form a derivate monophyletic group of endoparasitic species, some of which are of great medical and veterinary importance. For instance, the World Health Organization’s list of neglected tropical diseases (http://www.who.int/neglected_diseases/diseases/en/) includes several caused by endoparasitic flatworms, namely foodborne trematodiases (caused by *Clonorchis* spp. and *Fasciola* spp., among other flukes), schistosomiases (caused by *Schistosoma* spp.), echinococcoses (caused by *Echinococcus* spp.), and taeniases/cysticercosis (caused by *Taenia* spp.).

Tapeworms are obligate endoparasites of vertebrates that display complex life cycles in which morphologically and physiologically distinct forms alternate, adapted to the survival and development of different intermediate host species [4, 5]. The Cestoda class is divided into two subclasses: Eucestoda and Cestodaria. The Eucestoda subclass (‘true’ tapeworms) includes *Echinococcus* spp. and *Taenia* spp., which are the main species of medical and veterinary interest. Eucestodes have increased fertility due to metamerism (the serial repetition of body structures, or metameres) [6]. In eucestodes, metamerism is represented by the internal serial repetition of their reproductive organs, called proglottization [4, 6]. In the majority of eucestode evolutionary lineages, proglottization is associated with the external delimitation (segmentation) of proglottids, in a developmental process called strobilation [4, 5]. Eucestode strobilation occurs during the transition from the larval to adult stage and involves the repetitive generation of new proglottids in the base of the head (scolex), in the so-called neck region. Strobilation persists during the whole life of the adult worm, with each proglottid moving toward the posterior end as a new one is generated in the neck region. Strobilation allows adult cestodes to have larger numbers of hermaphroditic sexual organ sets, promoting both cross and self-fertilization and the frequency of egg-release by the progressive excision of gravid proglottids [4, 6].

In cestode evolution, the eucestodes constitute the most recently evolved subclass, and includes orders with different degrees of proglottization/strobilation [4]. The most ancestral eucestodes (those from the order Caryophyllidea) do not undergo proglottization and are non-segmented, similar to the other ancestral cestodes (i.e. those from the Cestodaria sublass). On the other hand, eucestodes of the order Spathebothriidea have proglottization without body segmentation, whereas others from more recently evolved orders (e.g. members of the order Cyclophyllidea, including the most relevant cestodes from an epidemiological point of view) undergo full strobilation (i.e. proglottization along external segmentation).

Thus, the evolution of strobilated tapeworms from non-segmented ancestors could be explained by two hypotheses: the initial loss of segmentation from an ancestral segmented lophotrochozoan, followed by the re-emergence of this process in more recent eucestode lineages in the form of proglottization/strobilation; or the independent evolution of proglottization/strobilation within the eucestode lineage, which gave rise to the present proglottized or fully strobilated orders. Therefore, cestodes are interesting subjects for evolutionary developmental studies [7], especially for those aiming to elucidate the evolutionary origins of developmental novelties related to strobilation. To achieve this, it is important to comparatively analyze cestode genomes and identify candidate genes related to these developmental processes.

So far little is known about strobilation and other developmental processes in cestodes at the molecular level, despite the relevance of such knowledge for both basic evolutionary developmental studies [7] and the identification of targets for new alternative drugs against cestode parasites [8–11]. To better understand cestode molecular biology, several species have been targeted by “omics” studies, including genomic, transcriptomic, and proteomic surveys. *Echinococcus granulosus, Echinococcus multilocularis*, *Hymenolepis microstoma*, and *Taenia solium* were the first cestodes to have their genomes completely sequenced [12]. Later, the 50 Helminth Genomes Project provided the draft genome sequence of another 14 cestode species, along with those of many other helminths [13]. Furthermore, transcriptomic and proteomic data are available for different life-cycle stages of cestode species, such as *E. granulosus*, *E. multilocularis*, *H. microstoma*, and *Mesocestoides corti* [12, 14–18]. In some of these studies, the differential expression of transcripts and proteins between larval (non-strobilated) and adult (strobilated) forms has been assessed. For instance, tetrathyridia (larvae) and adult segmented worms of the *M. corti* species were compared with regard to their miRNA [14], mRNA [15, 16] and protein [17] repertoires, providing some clues about the gene products differentially expressed during the transition between these stages. The initial steps of strobilation were addressed by two proteomic studies; one comparing *M. corti* bona fide tetrathyridia with tetrathyridia after 24 h of strobilation induction [18] and the other identifying proteins newly synthesized in *E. granulosus* pre-adult forms (protoscoleces) upon strobilation induction [19].

Here, a data mining approach, integrating genomic and transcriptomic data, was carried out to identify cestode developmental genes and pathways, and to demonstrate the molecular mechanisms involved in the strobilation process. Eighteen species from the Protostomia clade of Bilateria metazoans were assessed; 10 strobilated and non-strobilated species of flatworms and 8 outgroup species. Their genome sequences and transcriptional profiles were compared to identify tapeworm developmental genes associated with strobilation and, among these genes, those that had differential expression in the cestode pre-strobilated and strobilated stages. Genes associated with the strobilation process had their evolutionary histories investigated through phylogenetic and positive selection analyses. Moreover, functional enrichment provided further information on annotated gene products, and co-expression network analyses provided further information on the products of hypothetical genes. Overall, 34 proteins associated with strobilation were identified, providing evidence of the involvement of both conserved and novel cellular pathways in cestode strobilation.

## Results

### Phylogenomic analyses of strobilated and non-strobilated Platyhelminthes cestode species

A phylogenomic analysis was carried out with 10 neodermatan genomes, comprising 5 tapeworm species to represent strobilated platyhelmiths (*E. granulosus*, *E. multilocularis*, *H. microstoma*, *M. corti*, and *T. solium*); and by 5 fluke species to represent non-strobilated platyhelmithes (*Clonorchis sinensis*, *Schistosoma haematobium*, *Schistosoma japonicum*, *Schistosoma mansoni*, and *Opisthorchis viverrini*). An outgroup set of 8 genomes was used in the analysis, comprising 6 genomes from nematodes to represent non-segmented helminths (*Caenorhabditis elegans*, *Globodera pallida*, *Haemonchus contortus*, *Onchocerca volvulus*, *Strongyloides ratti*, and *Trichuris muris*); 1 genome from an annelid to represent a segmented protostome (*Helobdella robusta*); and 1 genome from a mollusk to represent a non-segmented protostome (*Lottia gigantea*).

**Fig. 1:**
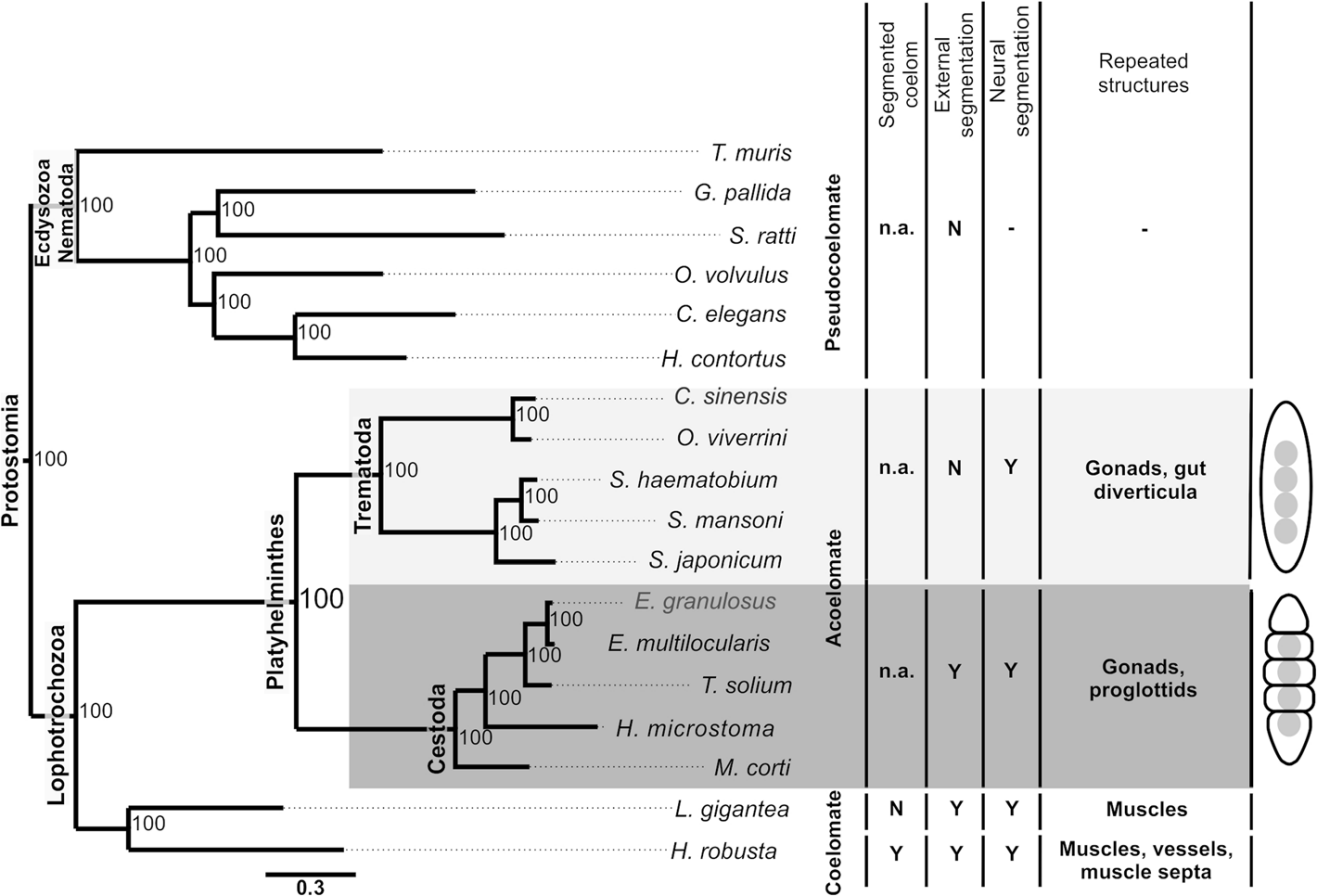
Platyhelminth evolutionary relationships and segmentation features. The phylogenomic tree (left) was built by MrBayes software with the VT + I + G evolutive model, for 1,688,000 generations, and with a set of 285 orthologs shared by all species. Platyhelminth species are highlighted, with the trematodes (flukes) shaded in light gray and the cestodes (tapeworms) shaded in dark gray. The numbers at the branches are Bayesian posterior probability values. Acelomated (platyhelminths), pseudocoelomated (nematodes), and coelomated (mollusk and annelid) species and corresponding segmentation features are indicated: external segmentation refers to segmented external structures derived from the epidermis (e.g. proglottids in cestodes); neural segmentation refers to ganglia repetition along the longitudinal axis (e.g. the “ladder-like” nervous system of cestodes); segmented structures refer to repeated organs or other anatomical features derived from the mesoderm (e.g. the repeated gonads in cestodes). Cartoons (right) illustrate the metamerism in flukes and full strobilation in tapeworms. Y = yes; N = no; n.a. = not applicable

Overall, 11,300 orthogroups of deduced protein sequences were identified, of which 285 have orthologous genes in all 18 analyzed species. These 285 protein sets were aligned, and the alignments were concatenated in a supermatrix for phylogenomic inference via Bayesian analysis. In the resulting tree (Fig. 1), two endoparasitic flatworm monophyletic groups were highly supported, with posterior probability of 100, one corresponding to flukes (trematodes) and the other to tapeworms (cestodes). The platyhelminthes were clearly divided into two clades, one with external body segmentation (with full strobilation) and the other with only internal segmentation (proglottization). Regarding protostome relationships, the tree supports the monophyly of the Ecdysozoa, Lophotrochozoa, Platyhelminthes, Cestoda, and Trematoda clades.

### Identification of strobilation-related proteins

**Fig. 2:**
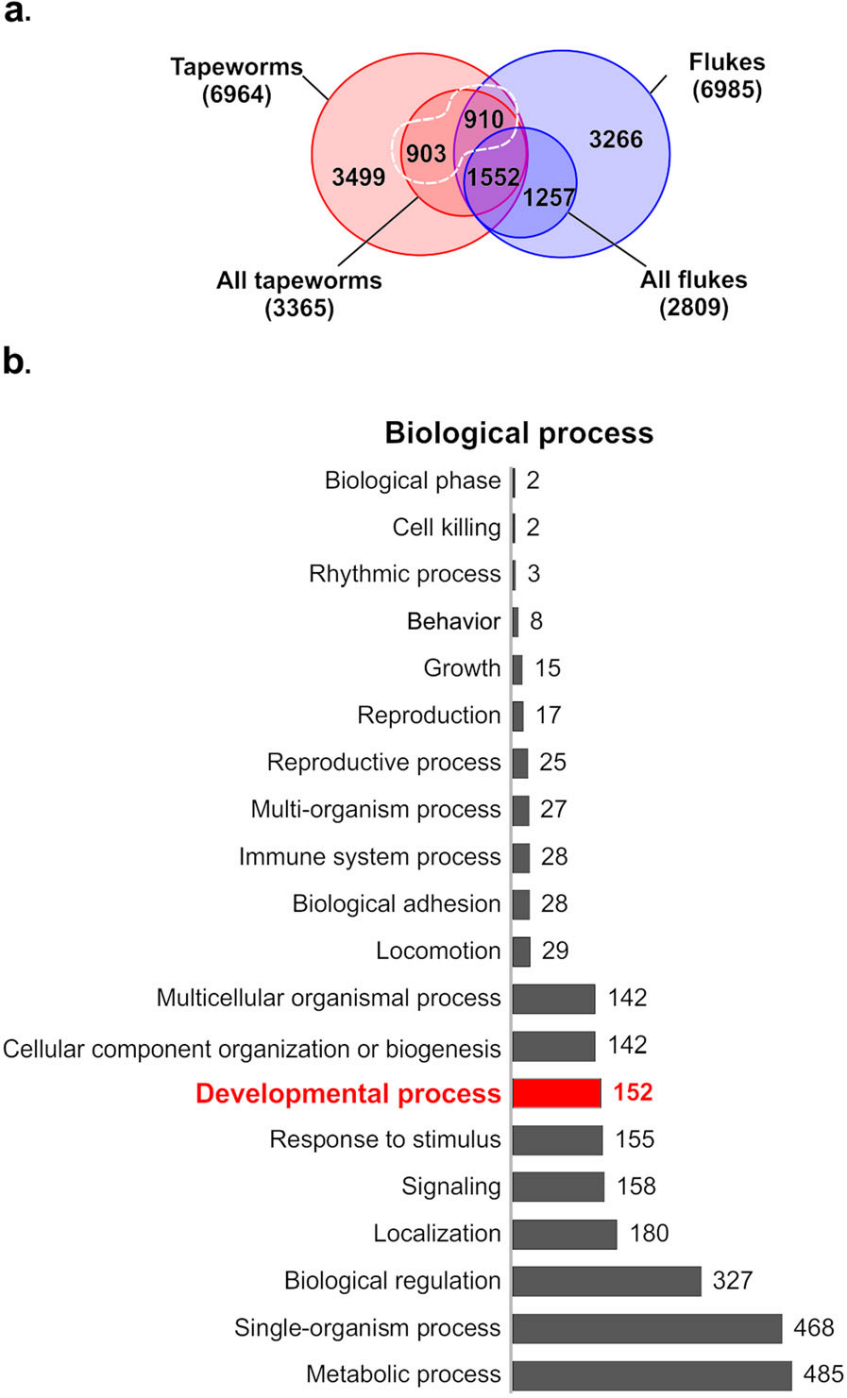
Summary of tapeworm and fluke orthogroups and functional enrichment of the tapeworm orthogroups selected as strobilation-related. **a** Venn diagram showing orthogroups shared between the sets of proteins from tapeworms (class Cestoda) and flukes (class Trematoda). The subsets of proteins found in all assessed species in each of these two classes are indicated. The 1813 selected tapeworm orthogroups, found in all strobilated species but absent from at least one non-strobilated species, are circled by a dashed white line; this set was formed of 910 orthogroups found in all tapeworms and absent in 1 to 4 of the assessed fluke species, along with 903 orthogroups present in all tapeworms and absent from all fluke species. **b** Biological processes to which the 1813 selected tapeworm orthogroups were assigned in the functional enrichment analysis. The bar lengths and the numbers indicate the total number of orthogroups assigned to each process, with the bar corresponding to the orthogroups assigned to ‘development process’ being highlighted in red

To identify proteins related to the strobilation process, orthogroups were selected based on their presence in tapeworm species or absence from fluke species (Fig. 2). From the 11,300 identified orthogroups of deduced protein sequences, 6964 (61.63%) were found in at least one tapeworm species, whereas 6985 (61.81%) were found in at least one fluke species (Fig. 2a). From this subtotal, a set of 3365 orthogroups were shared by all 5 tapeworm species, and a set of 2809 orthogroups were shared by all 5 fluke species (supplementary Figure S1). It was assumed that proteins essential for tapeworm development would be found in all strobilated species but may be absent from non-strobilated species. Based on this criterion, 1813 tapeworm strobilation-related orthogroups were initially selected (Fig. 2a; supplementary Table S1). From the 1813 selected orthogroups, 910 were found in all tapeworms that were absent from 1 to 4 fluke species, whereas 903 orthogroups were found in all tapeworms that were absent from all 5 fluke species.

As tapeworm strobilation is a developmental process, we performed a functional enrichment of the initial set of 1813 tapeworm strobilation-related orthogroups. The functional assignment of these orthogroups is shown in supplementary Table S1; the biological process assignment is summarized in Fig. 2b, and the assigned molecular functions and cellular components are summarized in supplementary Figure S2. Overall, 152 orthogroups were assigned to developmental processes (highlighted in yellow in supplementary Table S1) and then selected for further analyses. A total of 304 orthogroups of UF proteins (highlighted in blue in supplementary Table S1) were also selected, as at least some of these may also be related to developmental processes.

Furthermore, considering that the strobilation process occurs only in the adult tapeworm and not in the larval stage(s), we used available transcriptomic data of three tapeworm species (*E. multilocularis*, *H. microstoma,* and *M. corti*) to identify proteins from the selected 326 orthogroups (152 development-related orthogroups and 304 UF proteins) whose genes have differential expression in the larval (pre-strobilation) and adult (strobilated) stages. The differentially expressed orthologous genes in the larval and adult stages of the non strobilated flukes *S. haematobium* [20] and *S. mansoni* [21] were excluded to avoid genes not related to strobilation. This resulted in 12 development-related and 22 UF proteins (from now on identified as UF 1–22) being selected as strobilation-related proteins (Table 1, supplementary Table S2).

**Table 1:** Summary of the tapeworm proteins selected as being strobilation-related. The presence of orthologues in species of other taxa is indicated by an ‘x’. Differential expression of the corresponding genes in larval versus adult stages is indicated by an arrow, with up (↑) and down (↓) arrows indicating up and down-regulation, respectively, in the adult (strobilated) stage; a dot (●) indicates that there is no differential expression between these stages. Abbreviations of protein names are as follows: bone morphogenetic protein 2 (BMP-2); Cyclin-G-associated kinase (GAK); Groucho protein (Groucho); Homeobox protein B4a (HoxB4a); Lim homeobox protein lhx1 (LHX1); membrane-associated guanylate kinase protein 2 (MAGI2); serine:threonine protein kinase Mark2 (Mark2); atrial natriuretic peptide receptor 1 (NPR1); RNA binding motif single stranded interacting (RBMS); serine:threonine protein kinase (Ser:Thr kinase); mothers against decapentaplegic homolog 4-like (SMAD4); and Pangolin J (TCF/LCF). Proteins of unknown function (UF) are identified by numbers according to the corresponding orthogroups

Protein name	Platyhelminthes										Annelida	Mollusca		Nematoda				
	Trematoda					Cestoda												
	*C. sinensis*	*O. viverrini*	*S. haematobium*^a^	*S. japonicum*	*S. mansoni*^b^	*E. granulosus*	*E. multilocularis*^c^	*H. microstoma*^d^	*M. corti*^e^	*T. solium*	*H. robusta*	*L. gigantea*	*C. elegans*	*G. pallida*	*H. conturtus*	*O. volvulus*	*S. ratti*	*T.muris*
BMP-2						x	**↑**	**●**	**●**	x								
GAK		x	x	x	**●**	x	**↓**	**●**	**●**	x	x							
Groucho	x		**●**	x		x	**↓**	**●**	**●**	x	x	x	x			x		x
HoxB4a						x	**●**	**↓**	**●**	x								
LHX1						x	**↓**	**●**	**●**	x								
MAGI2						x	**↑**	**●**	**●**	x								
Mark2						x	**↓**	**●**	**●**	x	x	x	x			x	x	x
NPR1			x		**●**	x	**↑**	**●**	**●**	x		x						
RBMS						x	**↑**	**●**	**●**	x	x							
Ser:Thr kinase						x	**↓**	**●**	**●**	x	x	x						
SMAD4			**●**			x	**↓**	**●**	**●**	x	x	x		x		x	x	x
TCF/LCF						x	**↓**	**●**	**●**	x	x							
UF1		x				x	**↑**	**↑**	**●**	x								
UF2						x	**●**	**↑**	**●**	x								
UF3						x	**↑**	**●**	**↑**	x								
UF4						x	**↓**	**●**	**●**	x								
UF5						x	**↑**	**●**	**●**	x								
UF6						x	**●**	**●**	**↓**	x								
UF7						x	**↑**	**●**	**●**	x								
UF8						x	**↑**	**↑**	**●**	x								
UF9						x	**↑**	**●**	**●**	x								
UF10						x	**↓**	**●**	**●**	x								
UF11						x	**↓**	**●**	**●**	x								
UF12						x	**●**	**●**	**↑**	x								
UF13						x	**●**	**↑**	**●**	x								
UF14						x	**↑**	**↑**	**●**	x								
UF15	x	x			**●**	x	**↑**	**●**	**●**	x	x	x						
UF16			**●**			x	**↓**	**●**	**●**	x								
UF17						x	**↓**	**●**	**●**	x								
UF18	x	x	**↓**		**●**	x	**●**	**●**	**↑**	x								
UF19	x	x				x	**↑**	**●**	**●**	x								
UF20	x				**●**	x	**↑**	**●**	**↑**	x								
UF21		x				x	**↑**	**●**	**●**	x								
UF22						x	**↓**	**●**	**●**	x								

^a^*S. haematobium* expressed sequence tag libraries analysis (Young et al. 2012]) [20]

^b^*S. mansoni* RNA-seq analysis (Protasio et al. 2012) [21]

^c^*E. multilocularis* RNA-seq analysis (Tsai et al. 2013) [12]

^d^*H.microstoma* RNA-seq analysis (Tsai et al. 2013) [12]

^e^*M. corti* RNA-seq data analysis (Basika et al. 2019) [16]

**Fig. 3:**
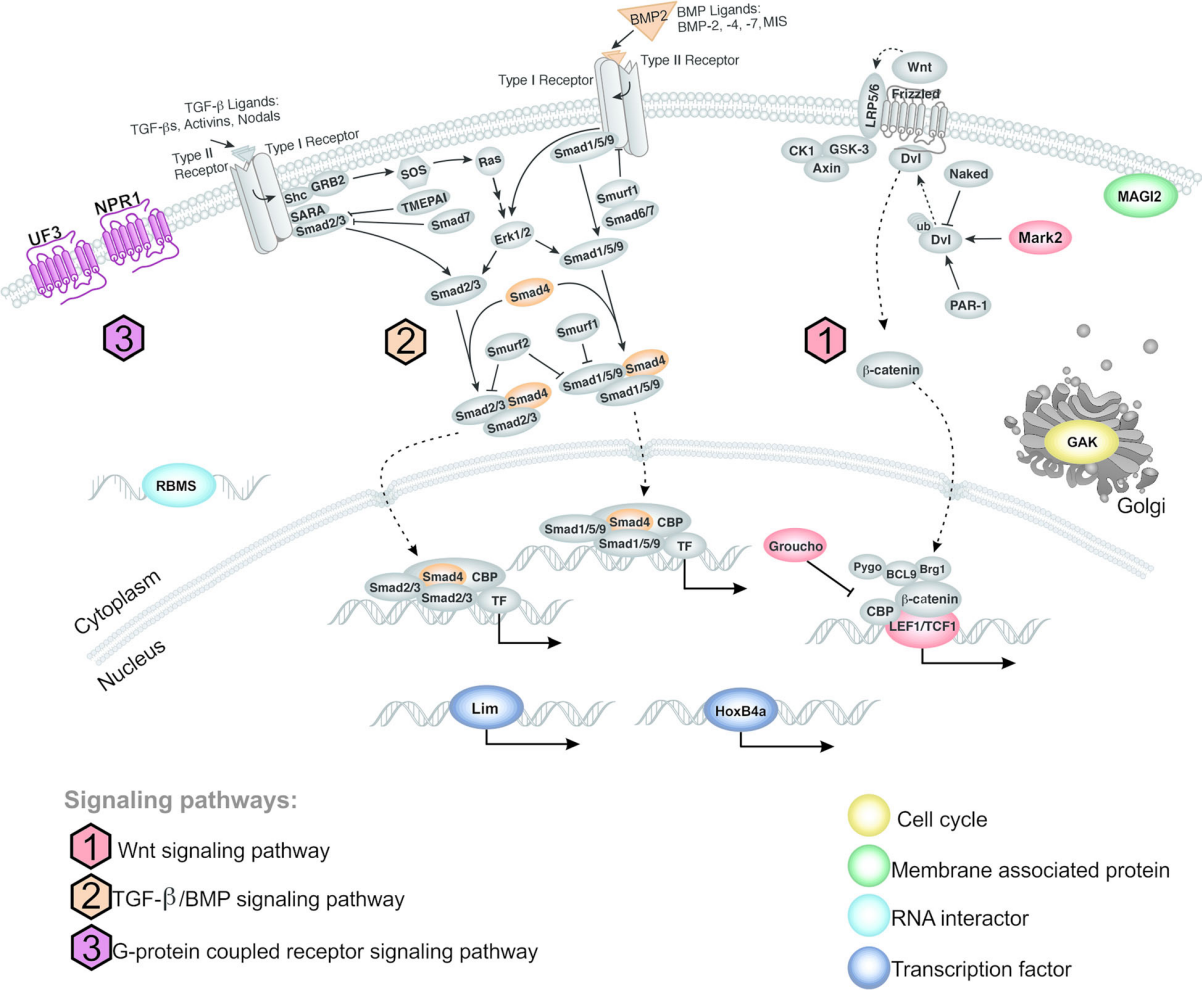
Schematic diagram showing cell pathways/functions to which the developmental proteins associated with strobilation were assigned. The set of 12 developmental proteins associated with cestode strobilation were mapped into different cell signaling pathways or assigned to different functional processes according to the KEGG database (https://www.genome.jp/kegg/). These proteins are indicated in different colors, whereas other functionally related cell proteins/structures/molecules are all shown in gray. The different signaling pathways are numbered as follows: 1 (red) for Wnt; 2 (orange) for TGF-β/BMP and 3 (purple) for G-protein coupled receptor signaling pathways. Other functional features are color-coded as follows: yellow for ‘cell cycle’; green for ‘membrane associated protein’; light blue for ‘RNA interactor’; and dark blue for ‘transcription factor’

The selected set of 12 proteins previously associated with development in other organisms was mapped into cellular pathways based on KEGG data (Fig. 3). Among these proteins, Groucho, MARK2, and TFC/LCF were mapped as components of the Wnt pathway; BMP2 and Smad4 were mapped as components of the TGF-β/BMP pathway; and NPR1 was mapped as component of the G-protein coupled receptor signaling pathway. This provided evidence of the involvement of some well-known developmental pathways in cestode strobilation.

**Fig. 4:**
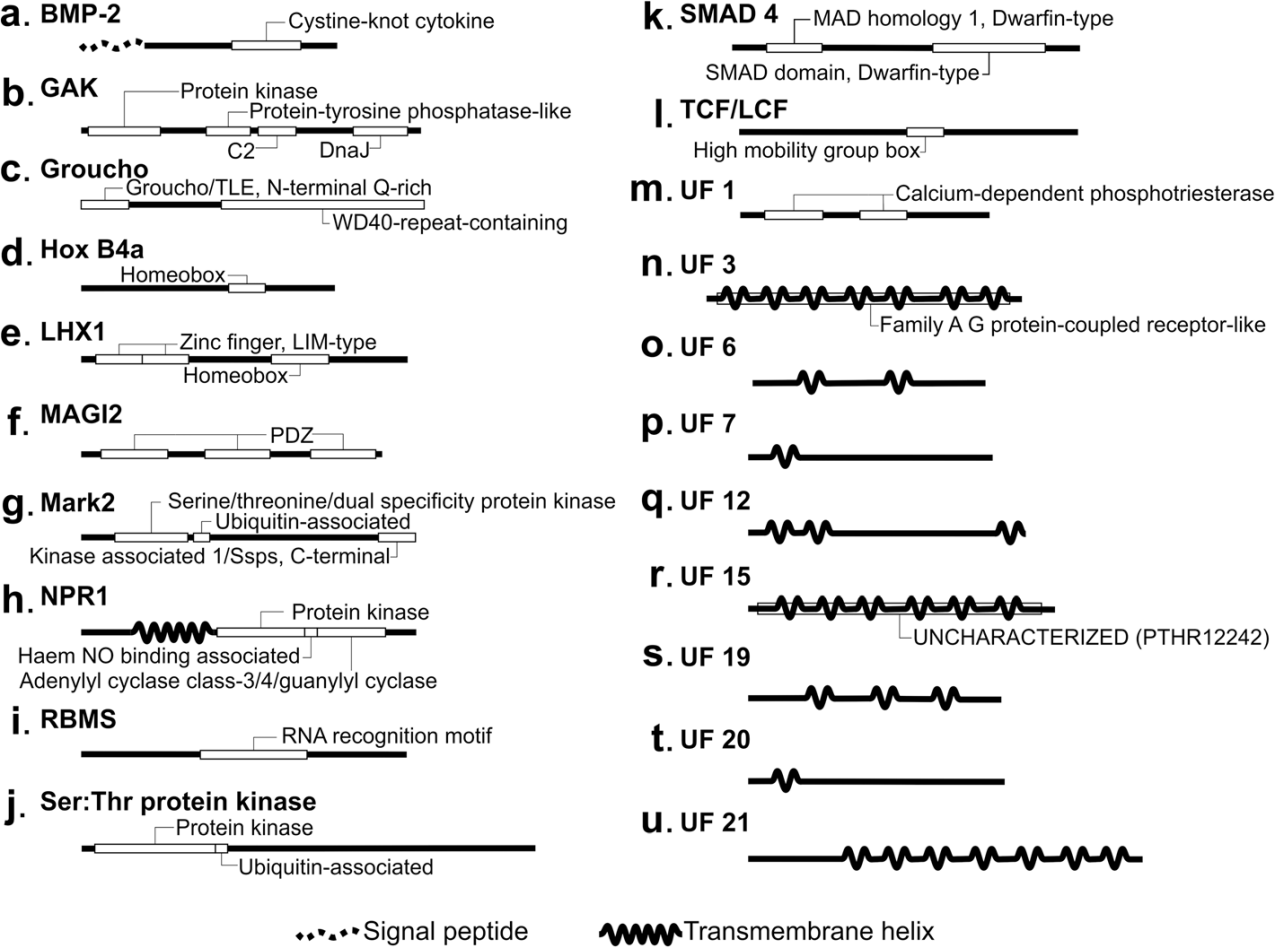
Domain profiles of the putative strobilation-related proteins. Schematics showing the domains shared by all tapeworm orthologues of (**a**) BMP2, (**b**) GAK, (**c**) Groucho, (**d**) HoxB4a, (**e**) LHX1, (**f**) MAGI2, (**g**) Mark2, (**h**) NPR1, (**i**) RBMS, (**j**) Ser:Thr kinase, (**k**) SMAD4, (**l**) TCF/LCF, (**m**) UF1, (**n**) UF3, (**o**) UF6, (**p**) UF7, (**q**) UF12, (**r**) UF15, (**s**) UF19, (**t**) UF20, and (**u**) UF21. Transmembrane helices are shown as a continuous wavy line, and signal peptides are shown as a dotted wavy line. Other domains are shown as white boxes with their names are indicated

To evaluate and confirm the homology of the proteins in the selected orthogroups, we performed functional domain predictions and comparisons (Fig. 4, supplementary Table S3). In all cases, the proteins within the orthogroup showed the same profile of predicted domains, further confirming their orthologies. In the set of 12 developmental proteins, several functional domains were identified, including a signal peptide and homeobox (Hox B4a and LHX1), RNA recognition motif, and PDZ domains. In the set of 22 UF proteins, 8 had transmembrane domains, two of them in association with another functional domain (a family A G protein-coupled receptor-like in UF 3 and an uncharacterized PTHR12242 domain in UF 15). Two calcium dependent phosphotriesterase domains were also identified in another UF protein (UF 1). No functional domains were found in the remaining 13 UF proteins.

### Evolutionary analyses of strobilation-related proteins

A comprehensive search for orthologs was carried out for all the identified strobilation-related proteins, defining 34 orthogroups. The resulting data is summarized in the supplementary Figure S3. It was verified that half (17) of these proteins (MAGI2, Mark2, RBMS, Ser:Thr kinase, TCF/LCF, UF 3, UF 5–14, and UF 22) had orthologs present only among cestodes. Only eight of the strobilation-related proteins had orthologs in non-flatworm lophotrocozoans, namely BMP-2, GAK, Groucho, Hox B4a, LHX1, NPR1, SMAD4, and UF 15. The remaining nine assessed targets (UF 1–2, UF 4, and UF 16–21) showed orthologs only among flatworms. It is interesting to note that considering this last set of 17 proteins were not cestode-exclusive, homologies among cestode orthologues (47.01–81.24%) were considerably higher than their homologies to orthologs from the other assessed taxa (15.82–48.45%) (supplementary Table S4).

Phylogenetic analyses were then performed to describe the evolutionary history of the 34 putative strobilation-related orthogroups (supplementary Table S5). The resulting trees (supplementary Figure S4–S37) agreed with the monophyly of tapeworms previously established by the phylogenomic analysis. The cyclophyllidean species from the Hymenolepididae and Mesocestoididae families (*H. microstoma* and *M. corti*, respectively) alternated as the most basal, whereas members of the Taeniidae family were more derived.

To address whether the evolution of these putative strobilation-related orthogroups could be under selective pressure, we performed searches to detect codons under positive selection. All codon sequences were aligned and the best phylogenetic tree for each orthogroup was selected (supplementary Table S6) for use in the positive selection analysis. The results suggested that the assessed orthogroups have not been under positive selection, except for a single site in the UF 16 orthogroup (supplementary Table S7).

### Functional analyses of strobilation-related UF proteins

Most of the proteins selected as being strobilation-related do not have any previously described biological function (UF 1–22). Therefore, to help determine their functional roles in tapeworm development, we performed a gene co-expression network analysis using the available transcriptomic data for the *E. multilocularis* pre-adult stage. Data from a pre-adult stage was used because of the lack of RNA-seq data from adult cestodes, in the amount and quality, required to allow the generation of a network based on gene co-expression.

**Fig. 5:**
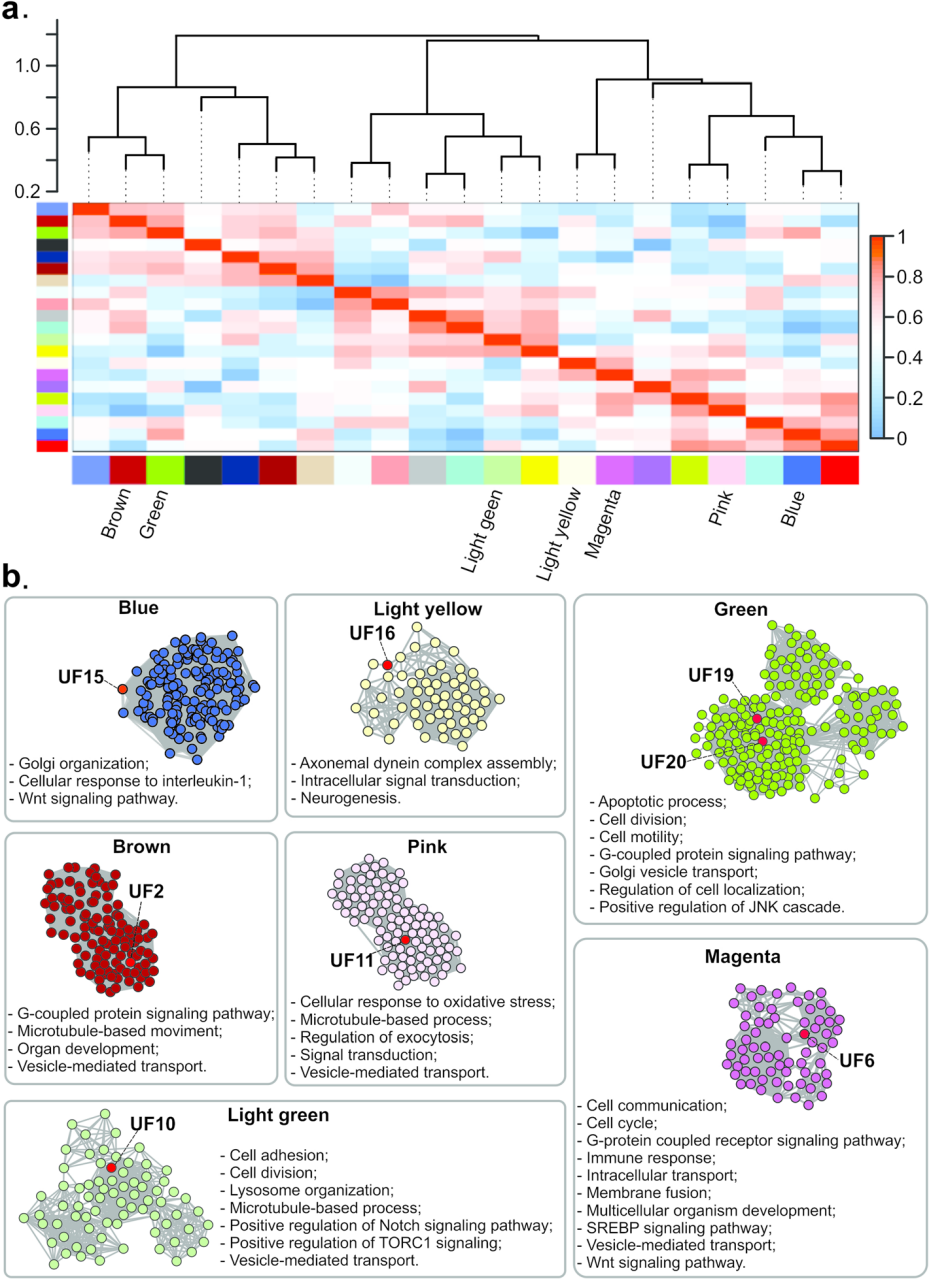
Analysis of gene co-expression networks and functional predictions for strobilation-related proteins of unknown function (UF). (**a**) Module relationships are represented by the dendrogram and the module Eigengene values of all module comparisons, as represented in the heatmap. Each identified module is represented by a different color and the 7 modules containing hub UF proteins are color-named. (**b**) Each box shows the results of the selected module of the corresponding color. UF proteins are named and highlighted in red in the module PPI networks. Functions predicted for each module are listed

Network construction was based on eight *E. multilocularis* RNA-seq samples, for which no sample outliers were identified (supplementary Figure S38). The resulting network consisted of 2957 nodes and 2,140,516 connections. Gene co-expressions were detected for 15 proteins in the set of 34 selected as strobilation-related, namely Hox B4a, LHX1, MAGI2, and NPR1 and UFs 2, 3, 5, 6, 10, 11, 13, 15, 16, 19, and 20. Only eight of them, namely UFs 2, 6, 10, 11, 15, 16, 19, and 20, were considered hubs (supplementary Table S8) and selected for further analyses. In Fig. 5, the modules to which the corresponding genes of these hub proteins belong to are indicated. With the aim of enhancing the information robustness of these selected network modules, we used the hub genes from each selected module as input for protein-protein interaction (PPI) analyses. Assuming that interacting proteins of a given module are usually involved in the same biological functions [22], we performed a functional enrichment for each of the eight selected modules (supplementary Figure S39 and supplementary Tables S9–15) to evaluate possible functions or pathways involving the strobilation-related UF proteins (Fig. 5). Based on the functional enrichment, the UFs 2, 6, 10, 11, 15, 16, 19, and 20 hub proteins were assigned to Wnt or G protein-coupled signaling pathways, and/or to biological functions such as vesicle-mediated transport, inductive cell migration, cell adhesion, apoptotic processes, and cellular response to interleukin-1, linking these pathways and functions to tapeworm strobilation.

## Discussion

Cestode developmental processes, including those involved in the transition from larvae to adult segmented worms (strobilation), are still poorly understood and a truly relevant theme in flatworm biology. Some previous proteomic and transcriptomic studies have started to unravel at least some of the molecular events underpinning cestode strobilation [12, 14–19], but we are still far from understanding all the mechanisms involved in this complex developmental process. In this study, we generated novel and complementary information regarding cestode strobilation using an integrative data mining approach that compared the genomic data of five tapeworm species and five fluke species, and compared the transcriptomic data of different developmental stages of three tapeworms and one fluke.

The draft genomes selected for analyses were chosen because of their advanced stages of sequencing, assembly, and annotation, and allowed the correct identification of genes/proteins and a precise definition of orthogroups. With that, a dependable assessment of the presence/absence of orthologues in the genomes of closely related tapeworm and fluke species was achieved. Overall, 34 proteins (12 developmental proteins and 22 UF proteins) were selected based on the following criteria (i) presence in all fully strobilated species; (ii) absence in at least one non-strobilated species; (iii) annotation as a developmental gene/protein or with unknown function; and (iv) differential expression in strobilated developmental stages.

The use of such an approach and criteria resulted in the identification of a set of strobilation-related genes that can be regarded as complementary to other sets of genes previously associated with this developmental process based solely on their differential expression at the transcriptional level [12, 14–16] and/or at the protein level [17–19]. As each of these in silico or wet lab approaches rely on different technologies and present different advantages and limitations, they complement each other in the assessment of complex biological processes, such as those involved in development or disease [23–25]. Therefore, the ongoing and progressive assessment of cestode strobilation by different in silico and wet lab approaches is expected to generate a more comprehensive picture of the molecules, mechanisms, and developmental pathways underlying this complex biological process.

An interesting feature of the novel set of 34 proteins identified as being related to strobilation was that half (17) of them were exclusive from tapeworms. Moreover, the remaining 17 proteins, although not exclusive, were considerably more conserved among cestodes (with identities 20.2–44.6% higher) than with their orthologs from other taxa, suggesting some degree of specialization of these proteins within the Cestoda class. As expected, these “non-exclusive” proteins are monophyletic for tapeworms, and nine of them had orthologs identified only for flatworm species, including tapeworms and flukes. Therefore, within the set of 34 proteins selected as being strobilation-related, 17 are assumed to be related to cestode-exclusive functions, whereas 9 are assumed to be associated with functions exclusive to the phylum Platyhelminthes. Further studies are necessary to determine the possible specialized functions of these proteins in tapeworm or flatworm biology.

In this set of 34 strobilation-related proteins, only 8 had orthologs found in non-Platyhelminth lophotrochozoans. The other 26 proteins were conserved only among platyhelminths (with 17 of them being conserved only among cestodes). This suggests that any ancestral lophotrochozoan segmentation mechanism [3] may have changed or gained complexity with the addition of gene/proteins during platyhelminth or cestode evolution. The phylogenetic analyses performed for these 34 strobilation-related proteins showed that their evolutionary history agrees with the currently accepted monophyletic origin of tapeworms [26].

It was interesting to evaluate the synonymous versus non-synonymous substitution rates regarding the set of strobilation-related proteins, as low variability is assumed for developmental genes due to the strong functional constraints they are submitted to [27]. According to the positive selection analyses, the assessed sequences did not show statistically significant high ω values, indicating that they are probably under functional constraints. This is consistent with what is expected for genes involved in development and other vital processes [28, 29] (supplementary Table S7).

We also investigated whether any of the strobilation-related proteins were be under positive selection, considering that at least some of them could be potential targets for the development of novel drugs against flatworm parasites. A stronger positive selection may determine amino acid variations in ortholog proteins, even among closely related species. Therefore, this type of selection would be undesirable in proteins that are potential drug targets [29]. Variations in orthologs of related pathogenic species (for example, among tapeworm or fluke species) could indicate that some of these species would be responsive to the drug, whereas others would be less, or even not at all, responsive [30]. Among the analyzed proteins, there was only one site under positive selection in one protein (UF16). Therefore, almost all the proteins analyzed would be interesting potential targets for broad spectrum drugs in cestodiasis.

As most of these strobilation-related proteins had no functional annotation (22 UF proteins), we performed a systems biology analysis to identify functional modules with the aim of providing additional evidence of their involvement in developmental processes. For cestodes, an important limitation for this type of approach is the scarcity of adequate RNA-seq data, considering the need for large numbers of replicates with low variation among them. Fortunately, RNA-seq data available for the *E. multilocularis* pre-adult (protoscolex) stage matched all technical requirements related to the replicate number and data quality. This allowed us to allocate eight of the UF proteins, which are so far not characterized, as central nodes (hubs) from seven functional network modules. These seven modules were associated with biological functions such as cell signaling, apoptosis, cell adhesion, and transcriptional regulation. Unfortunately, functional assignments were not possible for the remaining 14 UF proteins with the available transcriptomic data. A possible explanation for this could be the absence, or low expression, of these strobilation-related genes/proteins in bona fide protoscoleces, a non-strobilated stage.

Domain analyses of UF proteins allowed the identification of the UF 3 protein as a putative G protein-coupled receptor and the identification of putative transmembrane regions for eight UF proteins, suggesting that these proteins may have strobilation-related functions on the cell surface. Interestingly, transmembrane domains were found in UF 6 (related to the G-protein coupled receptor, SREBP and Wnt signaling pathways), UF 15 (related to the Wnt signaling pathway), UF 19 and UF 20 (related to the G-protein coupled signaling pathway). Additionally, co-expression and PPI analyses allowed the association of several UF proteins with important cell signaling pathways, namely Wnt (UF 6 and UF 15), G-protein coupled receptor signaling (UF 2, UF 6, UF 19, and UF 20), Notch (UF 10), SREBP (UF 6), and TORC1 (UF 10). These data allow us to infer that these UF proteins play strobilation-related functions in cell-cell interactions mediated by one of these signaling pathways. The involvement of selected UF proteins with strobilation is suggested based on evidence from a more recent proteomic survey carried out by our group with *M. corti* (Camargo de Lima, J., Floriani, M.A., Debarba, J.A., Monteiro, K.M., Moura, H., Barr, J.R., Zaha, A., Ferreira, H.B., personal communication). This study showed that UF 16 is among the proteins that are newly synthesized within 24 h of strobilation induction.

Among the strobilation-related proteins with functional annotation, Groucho, Mark2, and TCF/LCF are components of the Wnt signaling pathway. As discussed above, two UF proteins, UF 6 and UF 15, were also assigned to the same PPI network that includes this pathway. The Wnt pathway is well conserved among metazoans [31] and has already been associated with several different developmental events in diverse organisms. Among flatworms, Wnt signaling was initially involved in planarian anterior-posterior axis (head/tail) specification during regeneration [32]. Moreover, in *E. multilocularis*, Wnt signaling inhibition has been related to protoscolex generation and specification of the primary (antero-posterior) body axis during larval metamorphosis [5, 33]. Our data now provide evidence that this pathway is also required for cestode strobilation.

Evidence for the association of the TGF-β/BMP signaling pathway with strobilation arose from the identification of two of its components, BMP2 and SMAD4, among the selected set of strobilation-related proteins. In metazoans, TGF-β/BMP signaling, including the BMP protein signal and the Smad family of transcription factors, regulates a wide variety of cellular processes, such as proliferation, differentiation, adhesion, migration, and apoptosis [34] and developmental events, such as body axis formation and regeneration [35]. Moreover, in *E. granulosus,* Smad4 transcription factor is expressed in the metacestode (cystic larval stage) and in protoscoleces in a tissue-specific manner, with the highest transcript levels being found in activated (strobilation-induced) protoscoleces [36]. Taken together, all this evidence corroborates the contribution of the TGF-β/BMP signaling pathway in cestode strobilation.

The G-protein coupled signaling pathway also seems to be related to strobilation, based on the initial selection of the NPR1 receptor. Moreover, the UF 3 protein also exhibited the classical domains found in receptors of this pathway. In line with this, the G-protein coupled receptor signaling pathway has been shown to be involved in invertebrate developmental processes [37], including movement, development, reproduction, and plasticity of the whole worm and neuronal development in *E. granulosus* [38, 39]. It is also notable that serotoninergic G-protein coupled receptors have great high potential as drug targets for antiparasitic intervention [39]. However, in *E. granulosus* and other cestodes, many proteins of the G-protein coupled pathway remain unknown because of the considerable sequence divergence among the invertebrate orthologs [38]. Interestingly, based on the transcript co-expression and PPI analyses, we were able to relate UF 2, UF 6, UF 19, and UF 20 proteins to this pathway. Also, according to our differential expression analyses, NPR1, UF 2, UF 6, and UF19 are upregulated in the larval stage. In contrast, UF 3 and UF 20 are up-regulated in the adult stage, suggesting the involvement of the G-protein coupled pathway in different processes related to these developmental stages of cestodes.

Finally, transcript co-expression and systems biology analyses associated UF 2, UF 6, UF10, UF 11, UF 15, UF 19, and UF 20 proteins with vesicle-mediated, or Golgi vesicle, transport. In addition, the UF 15 protein seems to be part of the Golgi organization and has already been identified in a proteomic survey of *E. granulosus* extracellular vesicles (da Silva, E.D., Battistella, M.E., dos Santos, G.B., Monteiro, K.M., Cancela, M., Ferreira, H.B., Zaha, A., personal communication). Additionally, the selected GAK protein is involved in clathrin-dependent trafficking from the trans Golgi network [40]. Vesicle transport has been related to cell signaling during development, as it would facilitate the long-distance trafficking of signaling molecules [41]. Overall, these results suggest that vesicle transport may also be important in signaling pathways involved in cestode strobilation.

Since the the vital importance of developmental processes for parasite biology and reproduction, the identified proteins can be considered as targets for the development of novel therapeutic strategies in cestodiasis control. For instance, proteins such as enolase, histone deacetylases, pyruvate kinase, and serotoninergic G-protein coupled receptors have been proposed as targets for cestodiasis therapeutic interventions [9, 38, 42, 43]. Here, we have provided details of at least 12 developmental proteins with the potential to be targets for drug repositioning and the design of novel anthelmintic drugs. For instance, Mark2 and MAGI2 already have orthologs with available structural data that could be used for structural modeling, binding site prediction, and drug-protein molecular docking studies [44]. Likewise, the strobilation-related proteins identified as being cestode-exclusive could also constitute an interesting set of proteins for functional studies and for design-specific therapeutic approaches to cestodiases. Moreover, all proteins identified here as being related to strobilation have the potential to be molecular markers of cestode development, provided they are further characterized regarding their spatial and temporal expression patterns in a suitable model system, such as *M. corti* [45].

## Conclusion

In summary, we performed comprehensive evolutionary and functional analyses using plathyhelminth genomic and transcriptomic data to determine the relationships between segmented and non-segmented species. Such analyses allowed the successful identification of a set of 34 evolutionary conserved cestode proteins, with known (12 proteins) or unknown (22 proteins) function, as possible components of developmental pathways required for strobilation. Several of these proteins could be functionally assigned to cell signaling pathways like Wnt, TGF-β/BMP, and G-protein coupled receptor pathways, linking them to strobilation. Moreover, most of the strobilation-related UF proteins were exclusive of platyhelminths or cestodes, and they may act in specialized segmentation mechanisms that evolved and operate in these organisms. It was also found that virtually none of the 34 strobilation-related genes are under positive selection. These results provide further information on the molecular mechanisms and evolution of the cestode strobilation process. Our data also highlighted several proteins of interest for future functional studies as potential developmental markers and/or targets for the development of novel antihelminthic drugs.

## Methods

### Orthogroup identification

Genome data used for the identification of orthologous genes were obtained from the following public databases: National Center for Biotechnology Information (http://www.ncbi.nlm.nih.gov/), Sanger Institute (http://www.sanger.ac.uk/), SchistoDB (http://schistodb.net/schisto/), WormBase (http://www.wormbase.org/#012-34-5), and WormBase ParaSite (https://parasite.wormbase.org/species.html). The species used in this study were the tapeworms *E. granulosus*, *E. multilocularis*, *H. microstoma*, *M. corti*, and *T. solium*; the flukes *C. sinensis*, *S. haematobium*, *S. japonicum*, *S. mansoni*, and *O. viverrini*; the nematodes *C. elegans*, *G. pallida*, *H. contortus*, *O. volvulus*, *S. ratti,* and *T. muris*; the annelid *H. robusta*; and the mollusk *L. gigantea*. Database accession numbers and references of the respective genome sequences are available in the supplementary Table S16. The OrthoMCL algorithm v2.0.8 [46] was used with default parameters to identify groups of orthologs and paralogs, hereinafter called orthogroups, among the whole deduced proteomes of all 18 organisms listed above.

### Phylogenomic analysis

A Python (https://www.python.org/) script was developed to select, from the OrthoMCL output (section above), all orthogroups with at least a representative sequence for each of the 18 assessed organisms (supplementary data 1). If any given organism was found to have paralogous sequences, only the longest sequence was used for further analysis, keeping only one orthologous sequence for any given protein for each of the 18 organisms. The resulting multi-FASTA ortholog files for each protein sequence were used as the input for multiple alignments, performed using the CLUSTAL Omega algorithm [47] with default parameters. Subsequently, the SCaFos software [48] was used to concatenate the aligned files of amino acid sequences. The selection of supermatrix best-fit model of protein evolution was performed using ProtTest 3 [49]. The MrBayes v3.2.2 software [50] was used to construct the phylogenomic tree, with two runs, four chains in parallel, 25,000,000 generations, sampling every 100 generations, with a burn-in of 25%, and a stopval of 0.01 for the control of topological convergence.

### Identification of proteins associated with strobilated species or life-cycle stages

The orthogroups that had orthologous proteins in all five of the assessed cestode (strobilated) species, but lacked orthologs in at least one of the assessed trematode (non-strobilated) species, were selected using the Python script shown in supplementary data 2. Next, the resulting set of orthogroups associated with strobilated species were categorized and functionally enriched based on the BLAST sequence homologies and gene ontology (GO) annotations using the Blast2GO software [51]. The orthogroups were separated into two subsets: one with proteins of known functions and one with proteins of unknown functions (UF). Among the proteins in the known functions subset, we selected only the development-associated orthogroups.

Published transcriptomic data for *E. multilocularis, H. microstoma* [12], and *M. corti* [15] were used to identify proteins from selected orthogroups that had genes with different expressions between larval (pre-strobilated) and adult (strobilated) stages. Only bona fide larvae and adult worms were compared, as at present there is no RNA-Seq data available for specimens undergoing strobilation. To avoid differentially expressed genes that were not related to strobilation, those orthologs with differential expression between larval and adult stages of the flukes *S. haematobium* [20] and *S. mansoni* [21] were excluded from further analyses.

Considering the hypothesis that the segmentation mechanism underlying strobilation came from an ancestral lophotrochozoan mechanism [4], whether the selected strobilation-related proteins have orthologs in other species of the Lophotrocozoa superphylum was investigated. To achieve this, the proteins identified as being differentially expressed in the cestode larval and adult stages were used as query sequences in searches for additional orthologous sequences in other lophotrocozoan species. Searches were performed in the non-redundant database of NCBI-Genbank (https://www.ncbi.nlm.nih.gov/refseq/) using the blastp suite (https://blast.ncbi.nlm.nih.gov/Blast.cgi?PAGE=Proteins) and in the UniProtKB database (http://www.uniprot.org/) using the HMMER phmmer tool (https://www.ebi.ac.uk/Tools/hmmer/search/phmmer). Only orthologous sequences with identities and coverages greater than 30 and 70%, respectively, were selected. Functional domain annotation of orthologous proteins was performed using InterProScan 5 version 57.0 [52], and only those orthologous proteins in accordance with the functional domain profile of the orthogroup they were assigned to were used in further analyses.

### Phylogenetic and positive selection analyses

Multiple coding DNA sequences (CDSs) or deduced protein sequences were aligned using the CLUSTAL Omega algorithm [47] and the PRANK program [53]. CLUSTAL alignments were performed with default parameters, guided by an external hidden Markov model (HMM). PRANK alignments were performed using two algorithm variants, one based on an amino acid model (PRANK_AA_) and the other based on an empirical codon model (PRANK_C_). Nucleotide alignments were obtained using the PAL2NAL program [54]. Low quality regions of the generated sequence alignments were individually inspected, and manually adjusted, when possible, or removed, when necessary. The final nucleotide and amino acid alignments for all orthogroups were used in the phylogenetic analyses. Selection of the best-fit models of nucleotide and amino acid evolution was performed using the MEGA X software suite [55].

Phylogenetic trees were generated for each orthogroup by distance and probabilistic methods using the MEGA X software suite. For distance methods, the Neighbor-Joining algorithm was used, with pairwise deletion of gaps applied using the p-distance and Poisson evolutionary models for the amino acid sequence evaluation and the p-distance and Jukes-Cantor models for the nucleotide sequence evaluation. For the probabilistic method, the maximum likelihood algorithm with pairwise deletion of gaps was applied. The bootstrap tests of distance and probabilistic phylogenies were performed using 2000 repetitions for all analyses. Phylogenetic trees were also generated for each orthogroup by the bayesian method using the MrBayes v3.2.2 software, with two runs, four chains in parallel, 25,000,000 generations, sampling every 100 generations, with a burn-in of 25%, and a stopval of 0.01 for the control of topological convergence. The TreeView program [56] was used to visualize and edit the results of all generated phylogenies. The best phylogenetic tree estimated for each gene was selected based on its statistical support and on the agreement with the expected evolution for these species, as indicated by the phylogenomic tree.

Positive selection analyses were performed using the codeml program in the PAML 4 software [57]. The site-specific model analysis was implemented using the M0, M1a, M2a, M3, M7, and M8 nested models. For all models, a Bayes empirical Bayes (BEB) approach was employed to detect codons with a posterior probability of positive selection > 99% [58]. Positive selection is detected when ω > 1, which means non-synonymous substitutions rates (dN) are higher than synonymous substitutions rates (dS).

### Gene co-expression network analysis

Two RNA-seq datasets of the *E. multilocularis* pre-adult stage were used as inputs for the gene co-expression analyses. The first one was recovered from the ArrayExpress database https://www.ebi.ac.uk/arrayexpress/, accession number E-ERAD-50), and the second one was recovered from the Gene Expression Omnibus (GEO) database (https://www.ncbi.nlm.nih.gov/geo/, accession number GSE59173). Low quality reads were filtered using Trimmomatic v0.36 [59] and mapped on the *E. multilocularis* reference genome version 3 (ftp://ftp.sanger.ac.uk/pub/project/pathogens/Echinococcus/multilocularis/genome/), using the STAR 2.5.3a software [60]. The HTSeq package Version 0.8.0 [61], with intersection-nonempty counting parameter, was used to generate the gene count estimations. Expression data were imported into the R environment and normalized by a variance-stabilizing transformation method, available in the DESeq2 v1.18.1 package [62]. Finally, batch effect correction was performed utilizing the ComBat R package [63], implemented by the SampleNetwork R function created by Oldham et al. [64].

Gene co-expression calculation data was performed using the WGCNA R package, with the biweight midcorrelation method [65]. To provide a co-expression network topology close to a scale-free network, typical of many biological networks [66], a soft thresholding measurement was performed by raising co-expression values to a β power. The β power that generated a graph with the closest free-scale topology was selected. Next, a hierarchical clustering method, followed by a dynamical branch cutting algorithm, was chosen to identify modules. On each cluster, gene expression values were summarized into module eigengenes (ME). Intramodular hub genes (named as kME) were calculated by correlating the gene expression and the corresponding ME. Genes with kME values higher than 0.85 were considered as hub genes and were used as input to generate PPI networks via STRING (http://string-db.org) searches using the medium confidence interaction score (0.40) and excluding text mining source. For each module of interest, the PPI network generated by STRING was joined to the WGCNA co-expression network and only connections with a value > 0.4 were selected. The resulting co-expression and PPI networks were visualized using the Cytoscape software (v3.6.0) [67]. The sets of proteins in these networks were then used for the functional enrichment analyses using the Blast2GO software [51].

## Supplementary information

**Additional file 1.**

**Additional file 2.**

**Additional file 3.**

**Additional file 4.**

## Data Availability

All data and materials used in this research are publicly available. Genome web links are available in Supplementary Table S16. Transcriptomic data are available from https://www.ebi.ac.uk/arrayexpress/experiments/E-ERAD-50/ and https://www.ncbi.nlm.nih.gov/geo/query/acc.cgi?acc=GSE59173.

## Abbreviations

BEB: Bayes empirical Bayes
CDS: Coding DNA sequence
HMM: Hidden Markov model
ME: Module eigengenes
PPI: Protein-protein interaction
UF: Unknown function
