# Supplementary material for: Cestode strobilation: prediction of developmental genes and pathways
Source: BMC Genomics. 2020 Jul 16;21:487. doi: 10.1186/s12864-020-06878-3 (PMC7367335; doi:10.1186/s12864-020-06878-3)
Supplement: Supplementary file 2 — Additional file 2. [file 12864_2020_6878_MOESM2_ESM.docx]

Supplementary Figures

**
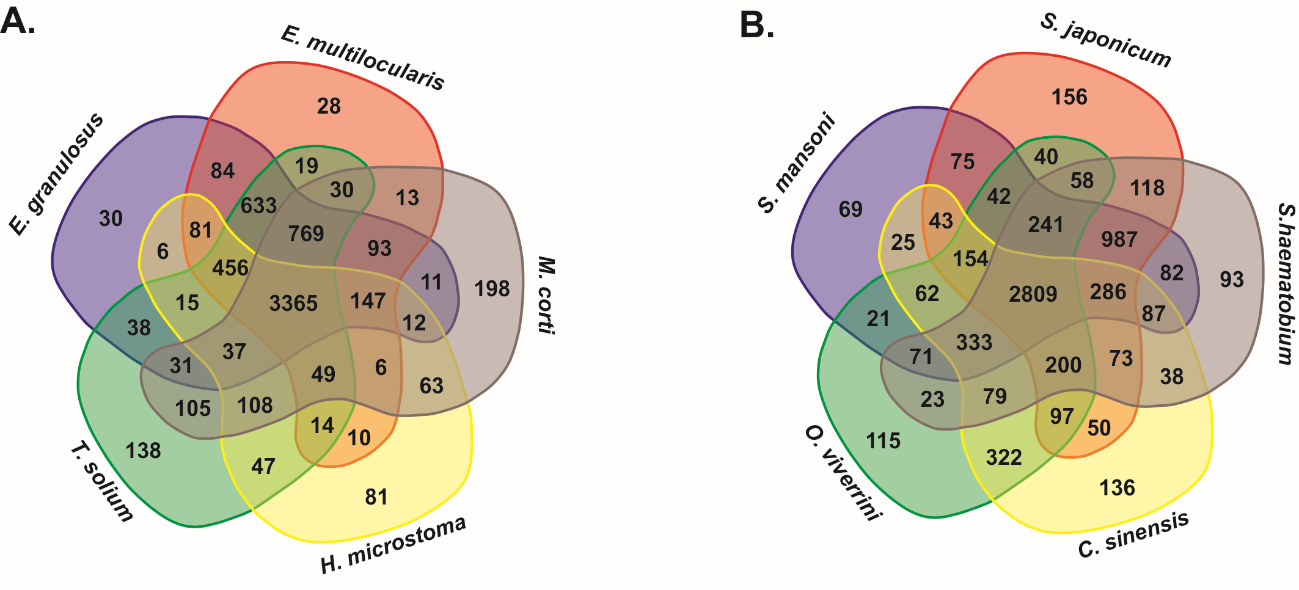
**

**Fig S1. Venn diagrams of tapeworm and fluke orthologues.** (A) Diagram showing orthogroups shared among the 5 assessed tapeworm species (*E. granulosus*, *E. multilocularis*, *H. microstoma*, *M. corti* and *T. solium*)*.* (B) Diagram showing orthogroups shared among the 5 assessed fluke species (*C. sinensis*, *O. viverrini*, *S. haemmatobium*, *S. japonicum* and *S. mansoni*).


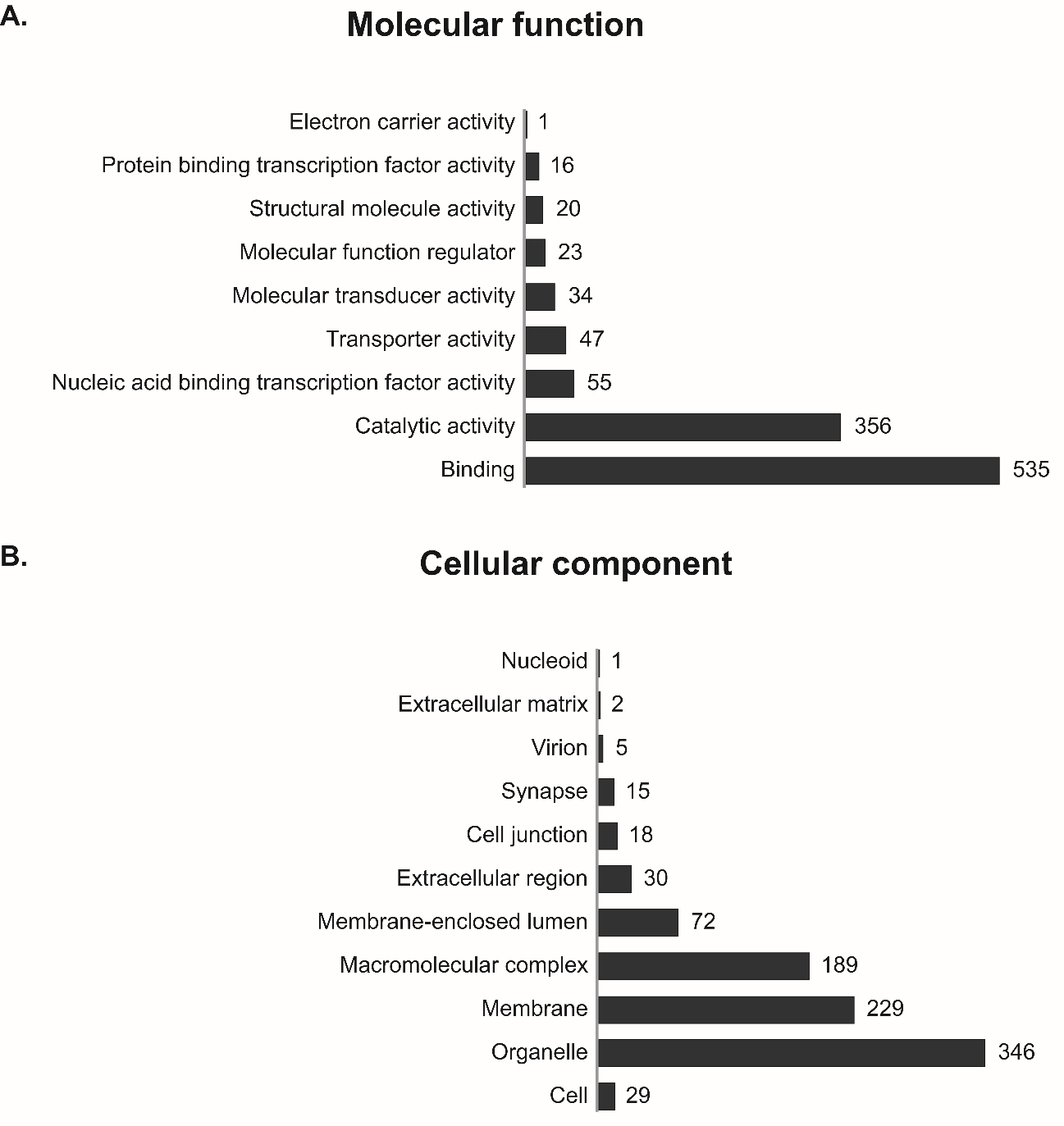


**Fig S2. Functional enrichment of orthogroups found in all assessed tapeworms and absent from at last one of the assessed flukes.** The bar graphics sumarizes the assignment of the 1813 selected orthogroups to (A) molecular functions, and (B) cellular components (see Figure 2).


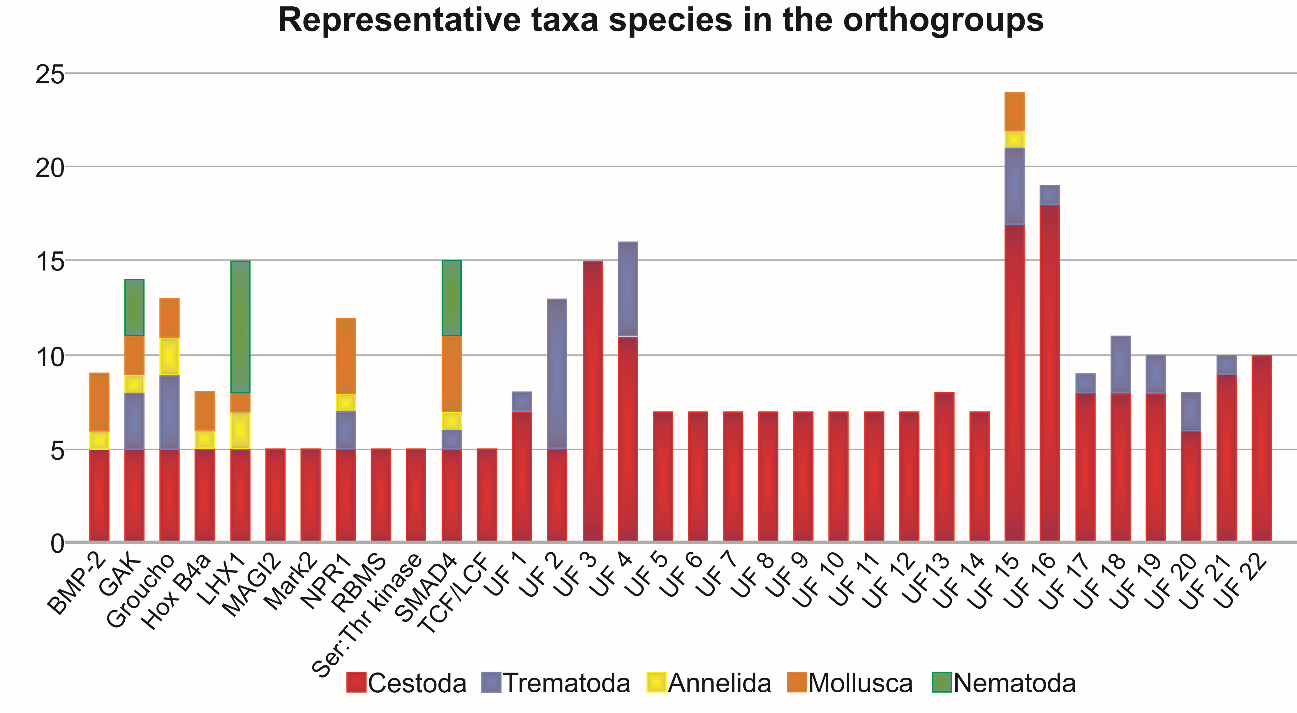


**Fig S3. Taxa representativeness in the strobilation-related orthogroups.** The graph shows the number of orthologs from each of the assessed taxa that were identified in each orthogroup.

**
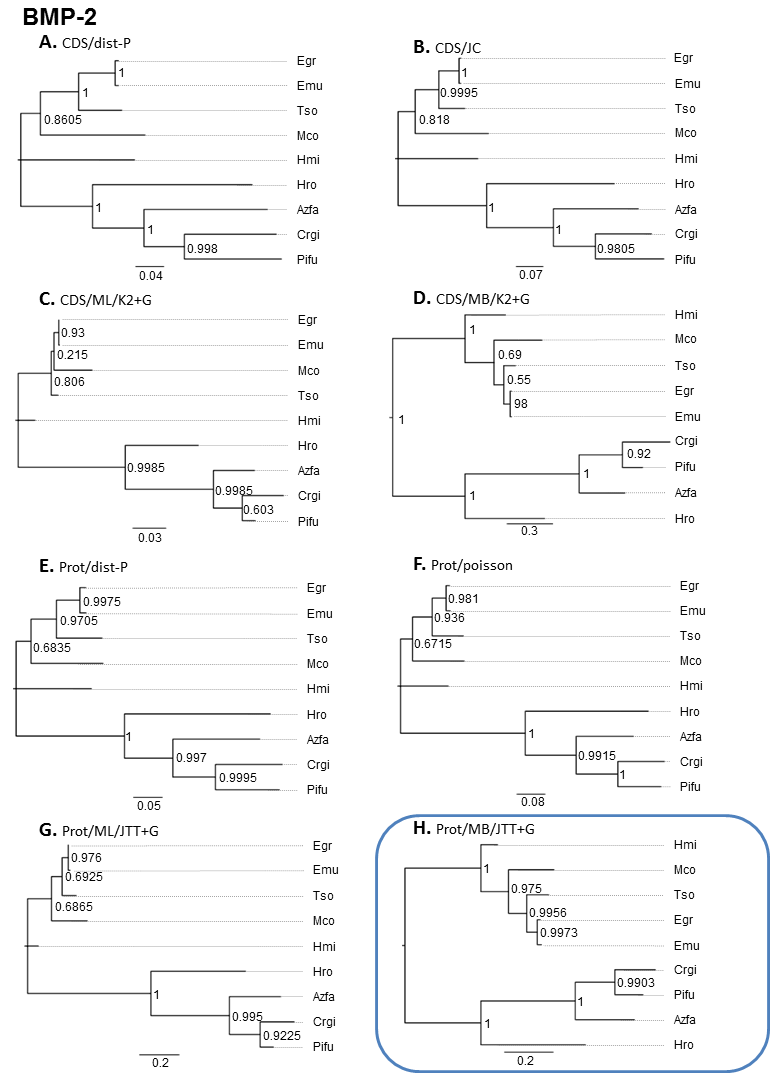
**

**Fig S4. Bone morphogenetic protein 2 (BNP-2) phylogenetic analysis.** (A-D) Phylogenetic trees based on CDS alignments; they were built using p-distance (A), Jukes-Cantor (B), maximum likelihood by K2 with gamma distribution (C), and bayesian by K2 with gamma distribution models (D). (E-H) Phylogenetic trees based on protein alignments; they were built using p-distance (E); Poisson (F), maximum likelihood by JTT with gamma distribution (G) and bayesian by JTT with gamma distribution models (H). The best phylogenetic tree is highlighted by a blue box. The analyzed species were Azfa, *Azumapecten farreri*; Crgi, *Crassostrea gigas*; Egr, *Echinococcus granulosus*; Emu, *Echinococcus multilocularis*; Hro, *Helobdella robusta*; Hmi, *Hymenolepis microstoma*; Mco, *Mesocestoides corti*; Pifu, *Pinctada fucata*; and Tso, *Taenia solium*. CDS and protein alignments are shown in Table S6.

**
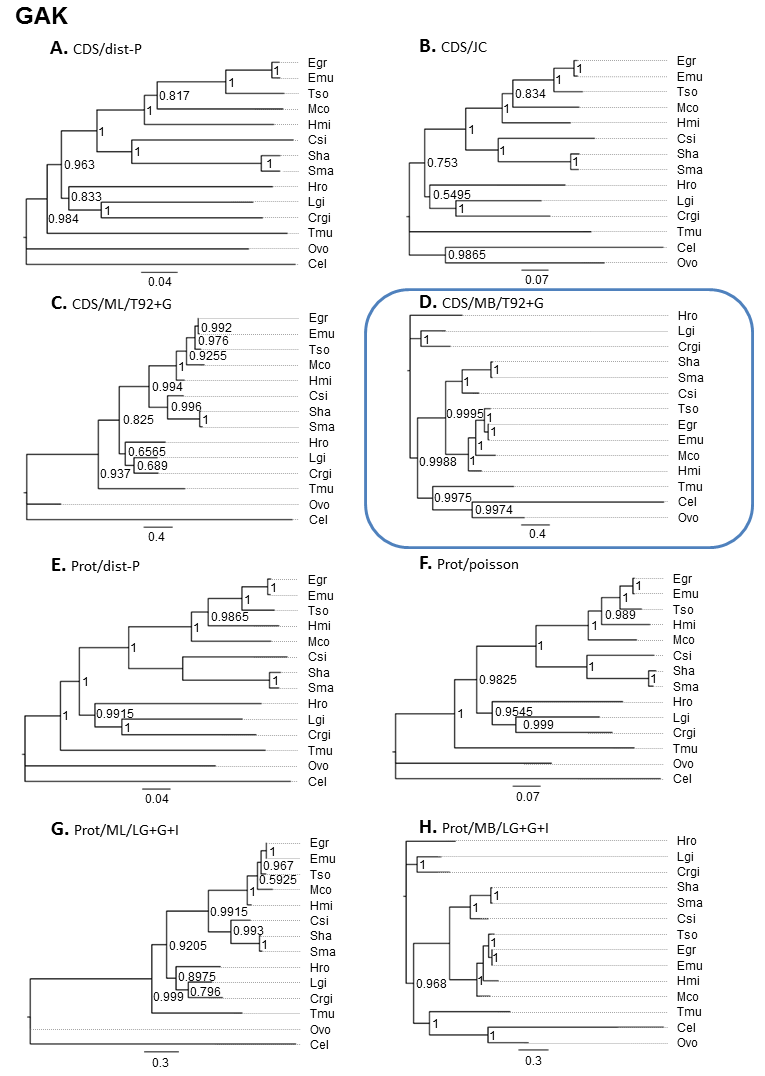
**

**Fig S5. Cyclin-g-associated kinase (GAK) phylogenetic analysis.** (A-D) Phylogenetic trees based on CDS alignments; they were built using p-distance (A), Jukes-Cantor (B), maximum likelihood by K2 with gamma distribution (C), and bayesian by K2 with gamma distribution models (D). (E-H) Phylogenetic trees based on protein alignments; they were built using p-distance (E); Poisson (F), maximum likelihood by JTT with gamma distribution (G) and bayesian by JTT with gamma distribution models (H). The best phylogenetic tree is highlighted by a blue box. The analyzed species were Cel, *Caenorhabditis elegans*; Csi, *Clonorchis sinensis*; Crgi, *Crassostrea gigas*; Egr, *Echinococcus granulosus*; Emu, *Echinococcus multilocularis*; Hro, *Helobdella robusta*; Hmi, *Hymenolepis microstoma*; Lgi, *Lollita gigantea*; Mco, *Mesocestoides corti*; Ovo, *Onchocerca volvulus*; Sha, *Schistosoma haematobium*; Sma, *Schistosoma mansoni*; Tso, *Taenia solium*; and Tmu, *Trichuris muris*. CDS and protein alignments are shown in Table S6.

**
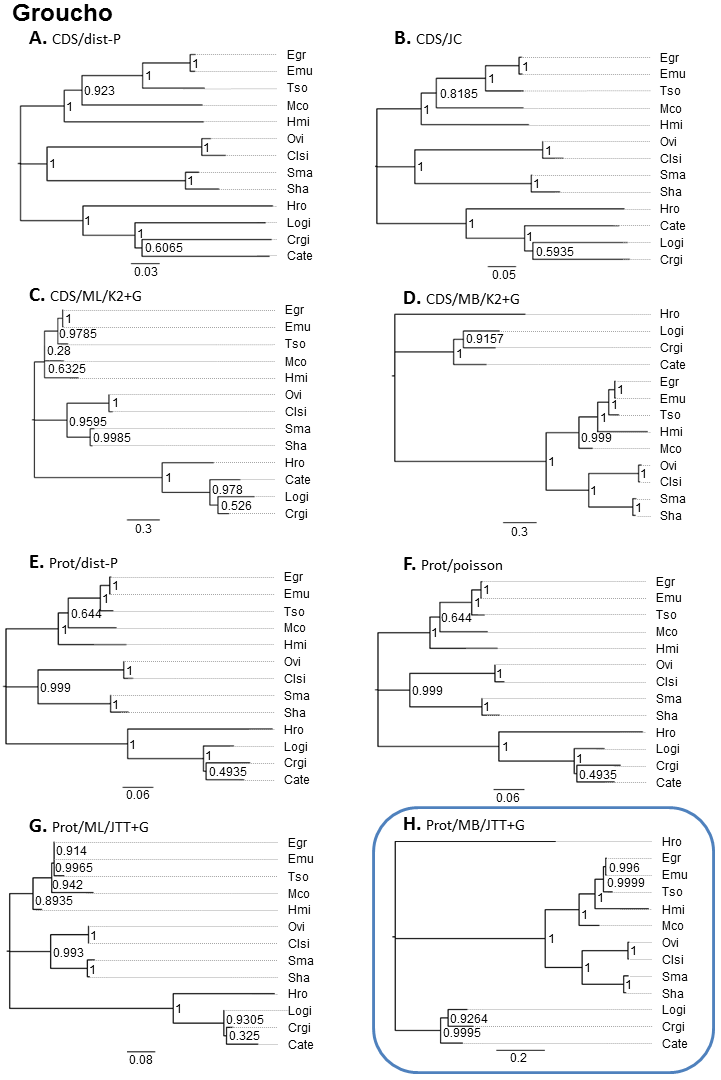
**

**Fig S6.** **Groucho protein phylogenetic analysis.** (A-D) Phylogenetic trees based on CDS alignments; they were built using p-distance (A), Jukes-Cantor (B), maximum likelihood by K2 with gamma distribution (C), and bayesian by K2 with gamma distribution models (D). (E-H) Phylogenetic trees based on protein alignments; they were built using p-distance (E); Poisson (F), maximum likelihood by JTT with gamma distribution (G) and bayesian by JTT with gamma distribution models (H). The best phylogenetic tree is highlighted by a blue box. The analyzed species were Cate, *Capitella teleta*; Csi, *Clonorchis sinensis*; Crgi, *Crassostrea gigas*; Egr, *Echinococcus granulosus*; Emu, *Echinococcus multilocularis*; Hro, *Helobdella robusta*; Hmi, *Hymenolepis microstoma*; Lgi, *Lottia gigantea*; Mco, *Mesocestoides corti*; Ovi, *Opisthorchis viverrini*; Sha, *Schistosoma haematobium*; Sma, *Schistosoma mansoni*; and Tso, *Taenia solium*. CDS and protein alignments are shown in Table S6.

**
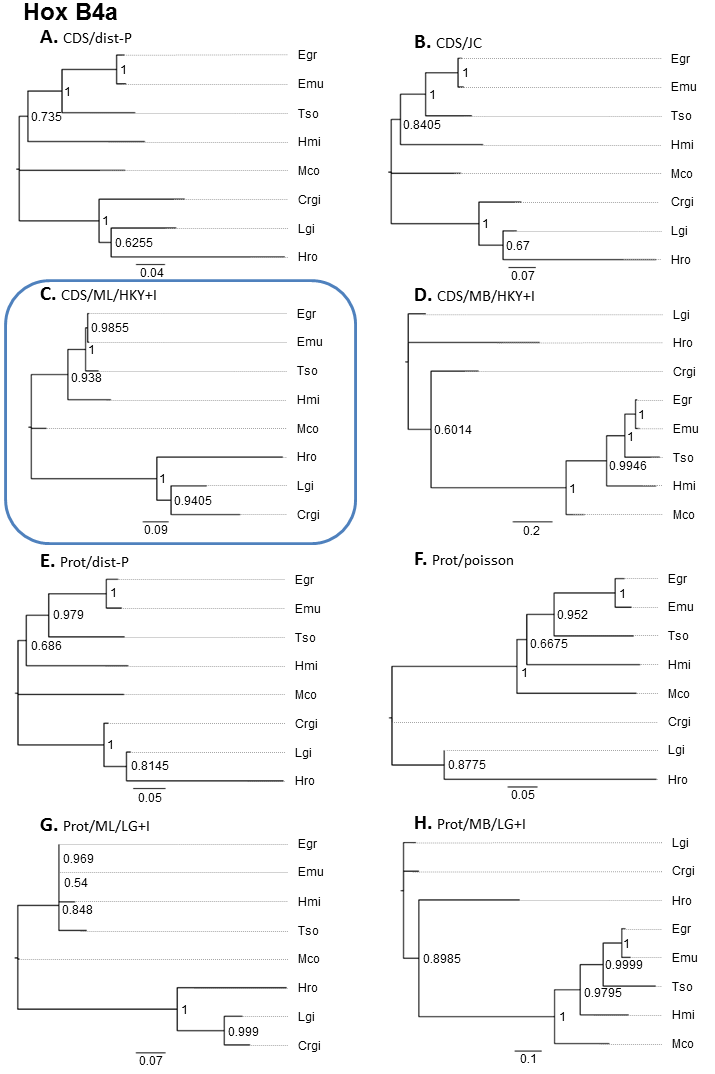
**

**Fig S7. Homeobox protein HoxB4a (Hox B4a) phylogenetic analysis.** (A-D) Phylogenetic trees based on CDS alignments; they were built using p-distance (A), Jukes-Cantor (B), maximum likelihood by K2 with gamma distribution (C), and bayesian by K2 with gamma distribution models (D). (E-H) Phylogenetic trees based on protein alignments; they were built using p-distance (E); Poisson (F), maximum likelihood by JTT with gamma distribution (G) and bayesian by JTT with gamma distribution models (H). The best phylogenetic tree is highlighted by a blue box. The analyzed species were Crgi, *Crassostrea gigas*; Egr, *Echinococcus granulosus*; Emu, *Echinococcus multilocularis*; Hro, *Helobdella robusta*; Hmi, *Hymenolepis microstoma*; Lgi, *Lollita gigantea*; Mco, *Mesocestoides corti*; and Tso, *Taenia solium*. CDS and protein alignments are shown in Table S6.

**
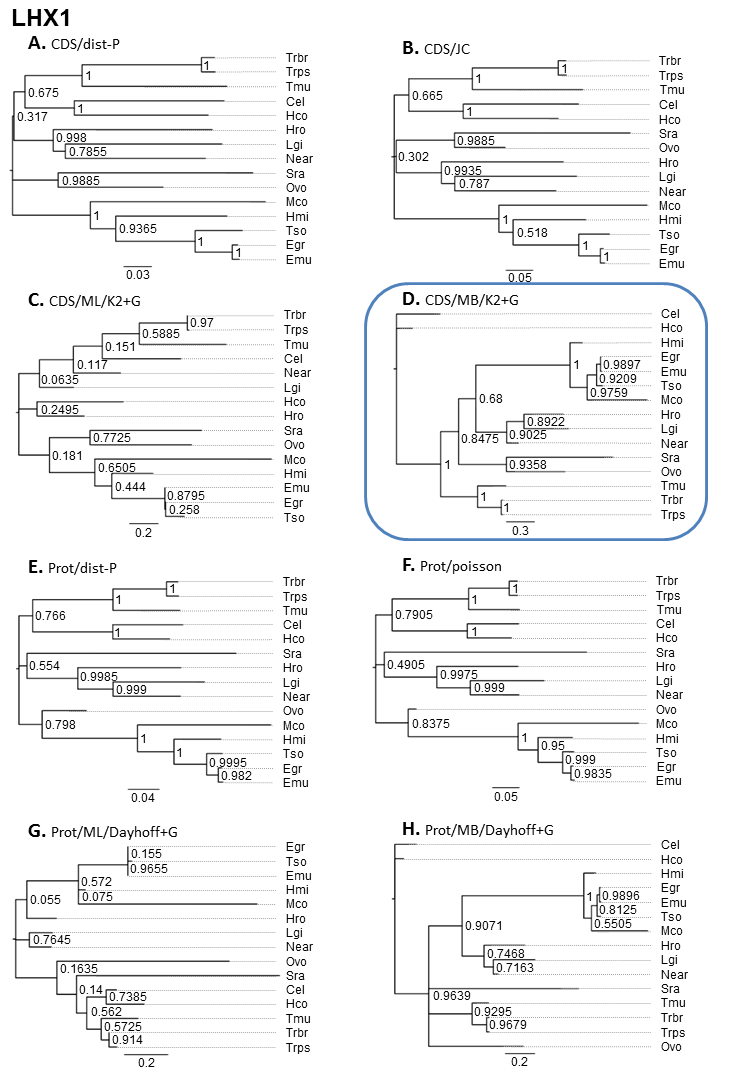
**

**Fig S8. Lim homeobox protein lhx1 (LHX1) phylogenetic analysis.** (A-D) Phylogenetic trees based on CDS alignments; they were built using p-distance (A), Jukes-Cantor (B), maximum likelihood by K2 with gamma distribution (C), and bayesian by K2 with gamma distribution models (D). (E-H) Phylogenetic trees based on protein alignments; they were built using p-distance (E); Poisson (F), maximum likelihood by JTT with gamma distribution (G) and bayesian by JTT with gamma distribution models (H). The best phylogenetic tree is highlighted by a blue box. The analyzed species were Cel, *Caenorhabditis elegans*; Egr, *Echinococcus granulosus*; Emu, *Echinococcus multilocularis*; Hco, *Haemonchus contortus*; Hro, *Helobdella robusta*; Hmi, *Hymenolepis microstoma*; Lgi, *Lollita gigantea*; Mco, *Mesocestoides corti*; Ovo, *Onchocerca volvulus*; Sra, *Strongyloides ratti*; Tso, *Taenia solium*; Trbr, *Trichinella britovi*; Trps, *Trichinella pseudospiralis*; Tmu, *Trichuris muris*; and Near, *Neanthes arenaceodentata*. CDS and protein alignments are shown in Table S6.

**
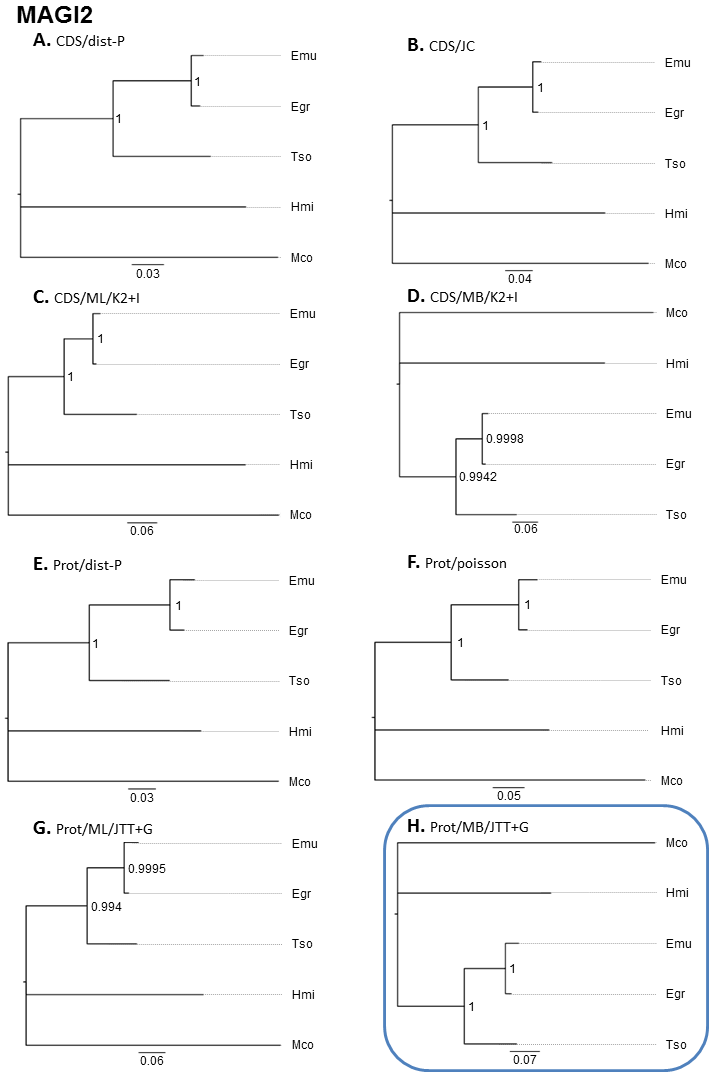
**

**Fig S9. Membrane-associated guanylate kinase protein 2 (MAGI2) phylogenetic analysis.** (A-D) Phylogenetic trees based on CDS alignments; they were built using p-distance (A), Jukes-Cantor (B), maximum likelihood by K2 with gamma distribution (C), and bayesian by K2 with gamma distribution models (D). (E-H) Phylogenetic trees based on protein alignments; they were built using p-distance (E); Poisson (F), maximum likelihood by JTT with gamma distribution (G) and bayesian by JTT with gamma distribution models (H). The best phylogenetic tree is highlighted by a blue box. The analyzed species were Egr, *Echinococcus granulosus*; Emu, *Echinococcus multilocularis*; Hmi, *Hymenolepis microstoma*; Mco, *Mesocestoides corti*; and Tso, *Taenia solium*. CDS and protein alignments are shown in Table S6.

**
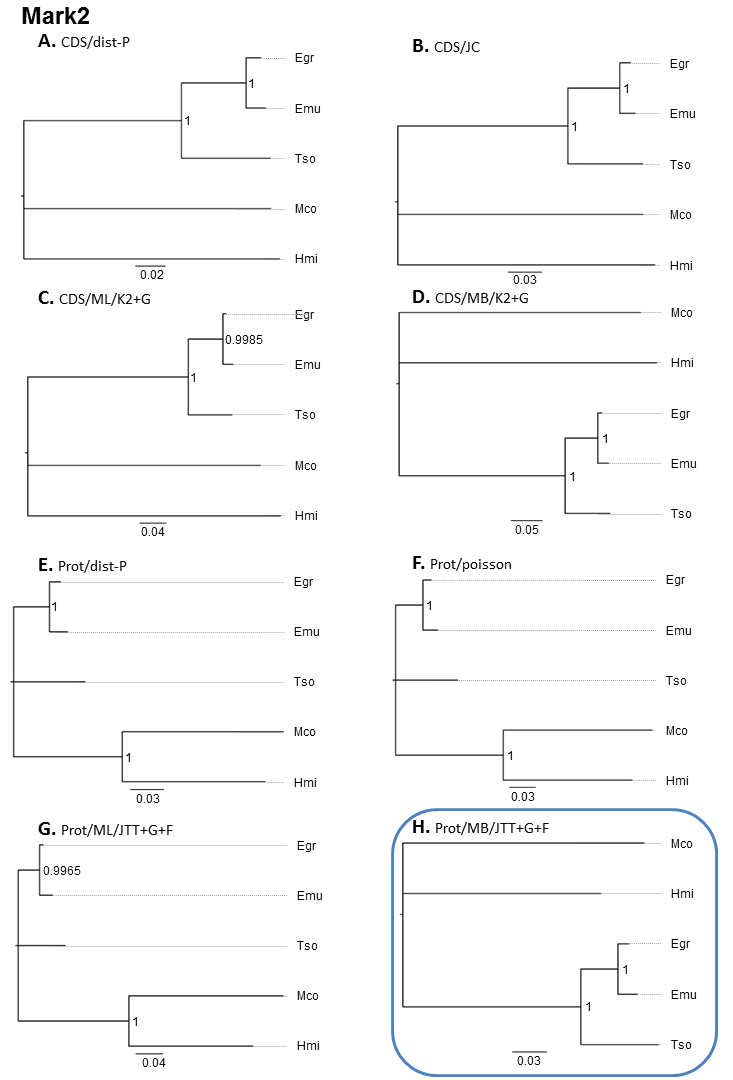
**

**Fig S10. Serine:threonine protein kinase Mark2 (Mark2) phylogenetic analysis.** (A-D) Phylogenetic trees based on CDS alignments; they were built using p-distance (A), Jukes-Cantor (B), maximum likelihood by K2 with gamma distribution (C), and bayesian by K2 with gamma distribution models (D). (E-H) Phylogenetic trees based on protein alignments; they were built using p-distance (E); Poisson (F), maximum likelihood by JTT with gamma distribution (G) and bayesian by JTT with gamma distribution models (H). The best phylogenetic tree is highlighted by a blue box. The analyzed species were Egr, *Echinococcus granulosus*; Emu, *Echinococcus multilocularis*; Hmi, *Hymenolepis microstoma*; Mco, *Mesocestoides corti*; and Tso, *Taenia solium*. CDS and protein alignments are shown in Table S6.

**
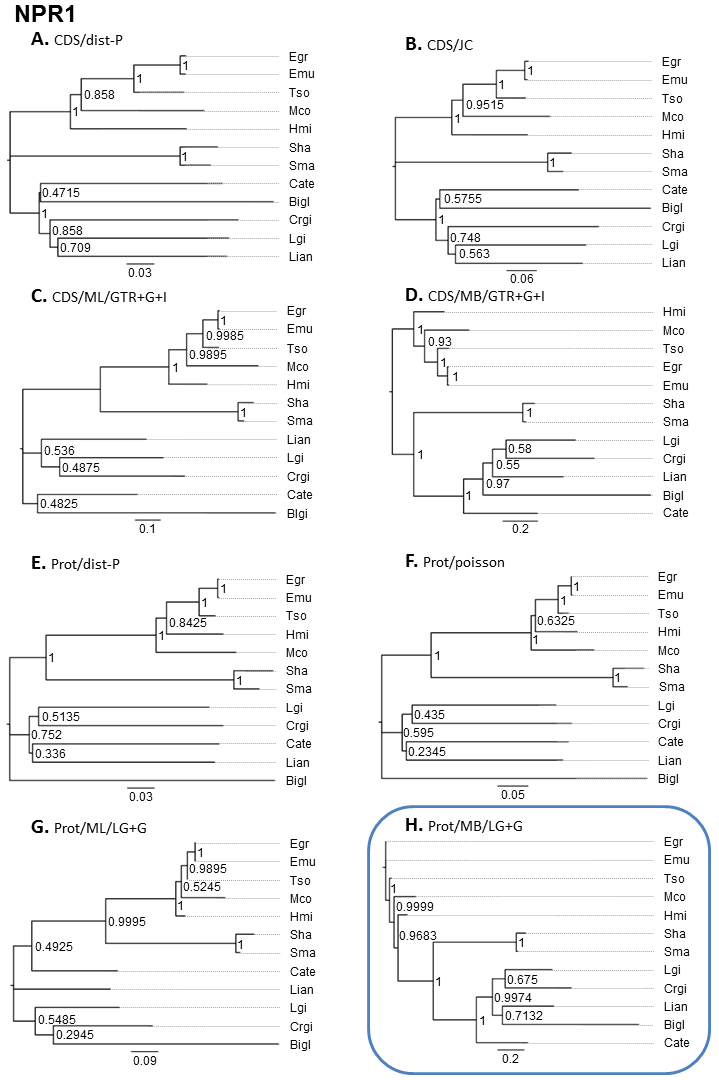
**

**Fig S11. Atrial natriuretic peptide receptor 1 (NPR1) phylogenetic analysis.** (A-D) Phylogenetic trees based on CDS alignments; they were built using p-distance (A), Jukes-Cantor (B), maximum likelihood by K2 with gamma distribution (C), and bayesian by K2 with gamma distribution models (D). (E-H) Phylogenetic trees based on protein alignments; they were built using p-distance (E); Poisson (F), maximum likelihood by JTT with gamma distribution (G) and bayesian by JTT with gamma distribution models (H). The best phylogenetic tree is highlighted by a blue box. The analyzed species were Bigl, *Biomphalaria glabrata*; Cate, *Capitella teleta*; Crgi, *Crassostrea gigas*; Egr, *Echinococcus granulosus*; Emu, *Echinococcus multilocularis*; Hmi, *Hymenolepis microstoma*; Lian, *Lingula anatine*; Lgi, *Lollita gigantea*; Mco, *Mesocestoides corti*; Sha, *Schistosoma haematobium*; Sma, *Schistosoma mansoni*; and Tso, *Taenia solium*. CDS and protein alignments are shown in Table S6.

**
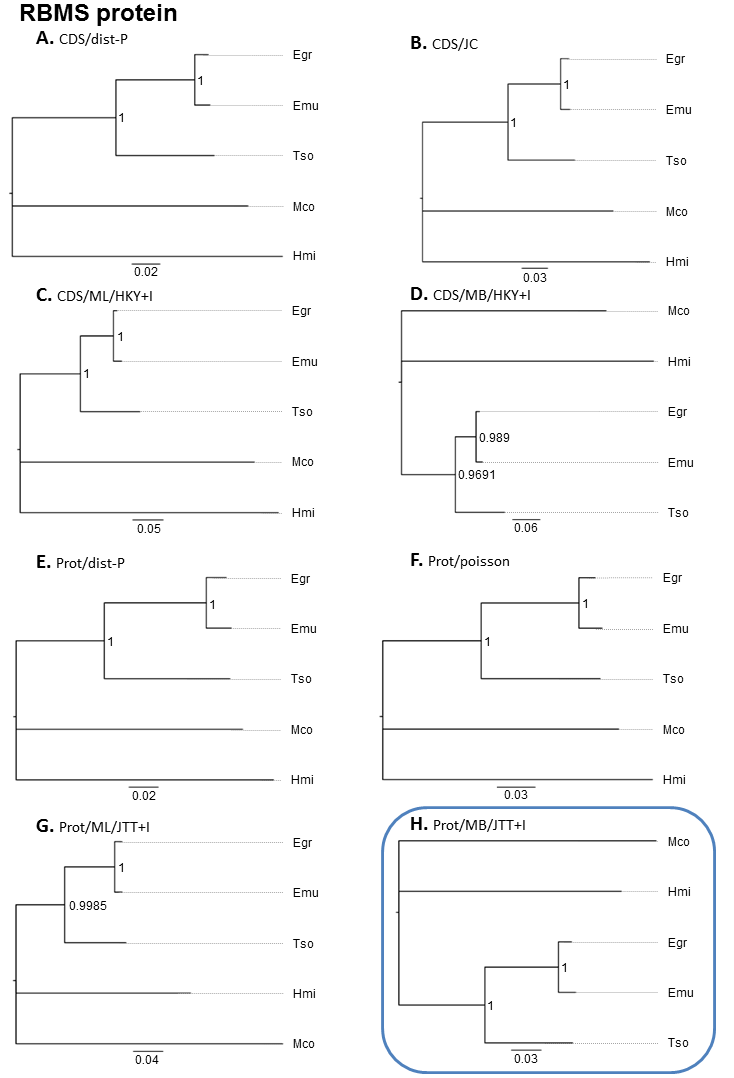
**

**Fig S12. RNA binding motif single stranded interacting (RBMS protein) phylogenetic analysis.** (A-D) Phylogenetic trees based on CDS alignments; they were built using p-distance (A), Jukes-Cantor (B), maximum likelihood by K2 with gamma distribution (C), and bayesian by K2 with gamma distribution models (D). (E-H) Phylogenetic trees based on protein alignments; they were built using p-distance (E); Poisson (F), maximum likelihood by JTT with gamma distribution (G) and bayesian by JTT with gamma distribution models (H). The best phylogenetic tree is highlighted by a blue box. The analyzed species were Egr, *Echinococcus granulosus*; Emu, *Echinococcus multilocularis*; Hmi, *Hymenolepis microstoma*; Mco, *Mesocestoides corti*; and Tso, *Taenia solium*. CDS and protein alignments are shown in Table S6.

**
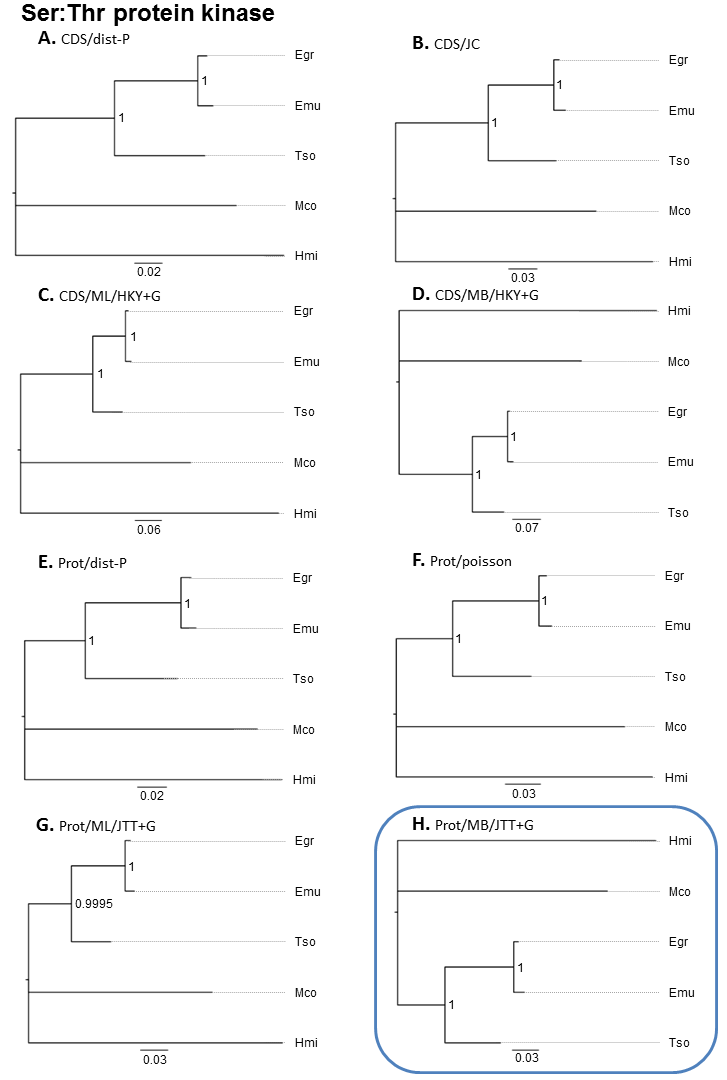
**

**Fig S13. Serine:threonine protein kinase (Ser:Thr protein kinase) phylogenetic analysis.** (A-D) Phylogenetic trees based on CDS alignments; they were built using p-distance (A), Jukes-Cantor (B), maximum likelihood by K2 with gamma distribution (C), and bayesian by K2 with gamma distribution models (D). (E-H) Phylogenetic trees based on protein alignments; they were built using p-distance (E); Poisson (F), maximum likelihood by JTT with gamma distribution (G) and bayesian by JTT with gamma distribution models (H). The best phylogenetic tree is highlighted by a blue box. The analyzed species were Egr, *Echinococcus granulosus*; Emu, *Echinococcus multilocularis*; Hmi, *Hymenolepis microstoma*; Mco, *Mesocestoides corti*; and Tso, *Taenia solium*. CDS and protein alignments are shown in Table S6.

**
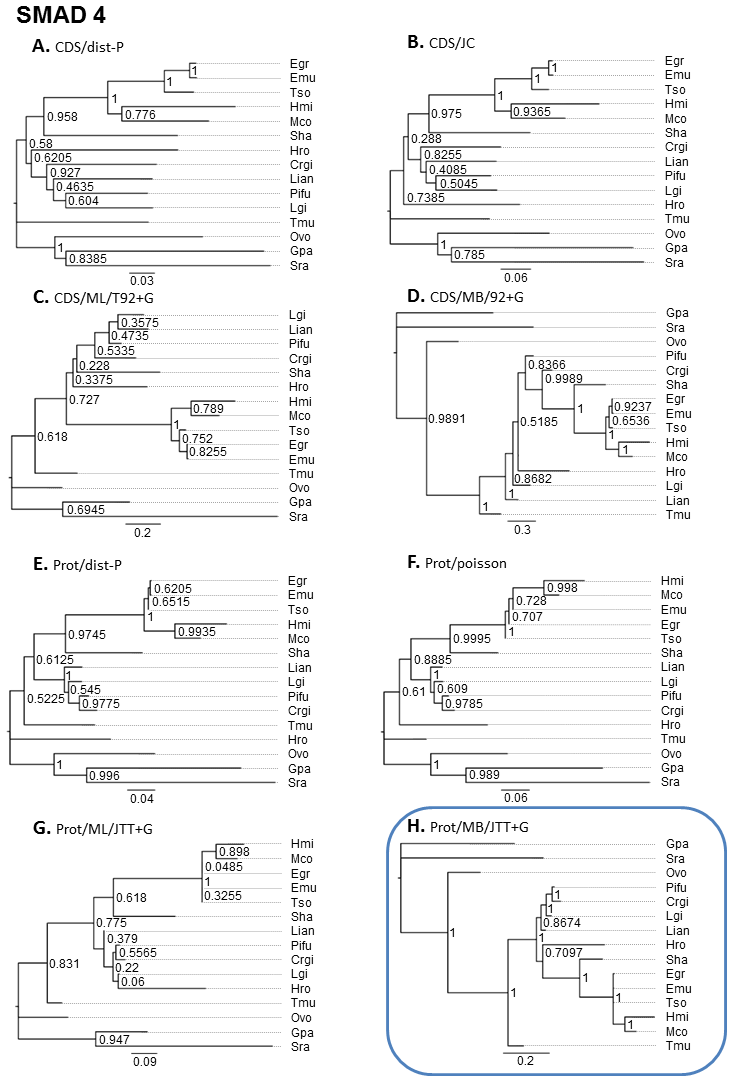
**

**Fig S14. Mothers against decapentaplegic homolog 4-like (SMAD 4) phylogenetic analysis.** (A-D) Phylogenetic trees based on CDS alignments; they were built using p-distance (A), Jukes-Cantor (B), maximum likelihood by K2 with gamma distribution (C), and bayesian by K2 with gamma distribution models (D). (E-H) Phylogenetic trees based on protein alignments; they were built using p-distance (E); Poisson (F), maximum likelihood by JTT with gamma distribution (G) and bayesian by JTT with gamma distribution models (H). The best phylogenetic tree is highlighted by a blue box. The analyzed species were Crgi, *Crassostrea gigas*; Egr, *Echinococcus granulosus*; Emu, *Echinococcus multilocularis*; Gpa, *Globodera pallida*; Hro, *Helobdella robusta*; Hmi, *Hymenolepis microstoma*; Lian, *Lingula anatina*; Lgi, *Lollita gigantea*; Mco, *Mesocestoides corti*; Ovo, *Onchocerca volvulus*; Pifu, *Pinctada fucata*; Sha, *Schistosoma haematobium*; Sra, *Strongyloides ratti*; Tso, *Taenia solium*; and Tmu, *Trichuris muris*. CDS and protein alignments are shown in Table S6.

**
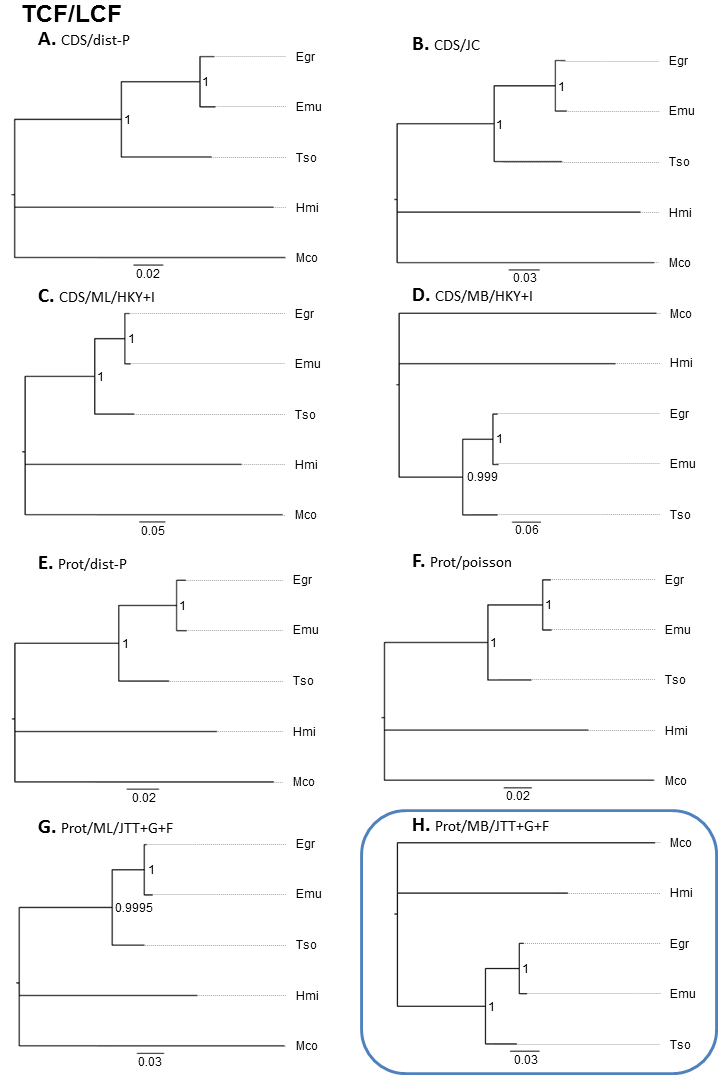
**

**Fig S15. Pangolin J (TCF/LCF) phylogenetic analysis.** (A-D) Phylogenetic trees based on CDS alignments; they were built using p-distance (A), Jukes-Cantor (B), maximum likelihood by K2 with gamma distribution (C), and bayesian by K2 with gamma distribution models (D). (E-H) Phylogenetic trees based on protein alignments; they were built using p-distance (E); Poisson (F), maximum likelihood by JTT with gamma distribution (G) and bayesian by JTT with gamma distribution models (H). The best phylogenetic tree is highlighted by a blue box. The analyzed species were Egr, *Echinococcus granulosus*; Emu, *Echinococcus multilocularis*; Hmi, *Hymenolepis microstoma*; Mco, *Mesocestoides corti*; and Tso, *Taenia solium*. CDS and protein alignments are shown in Table S6.

**
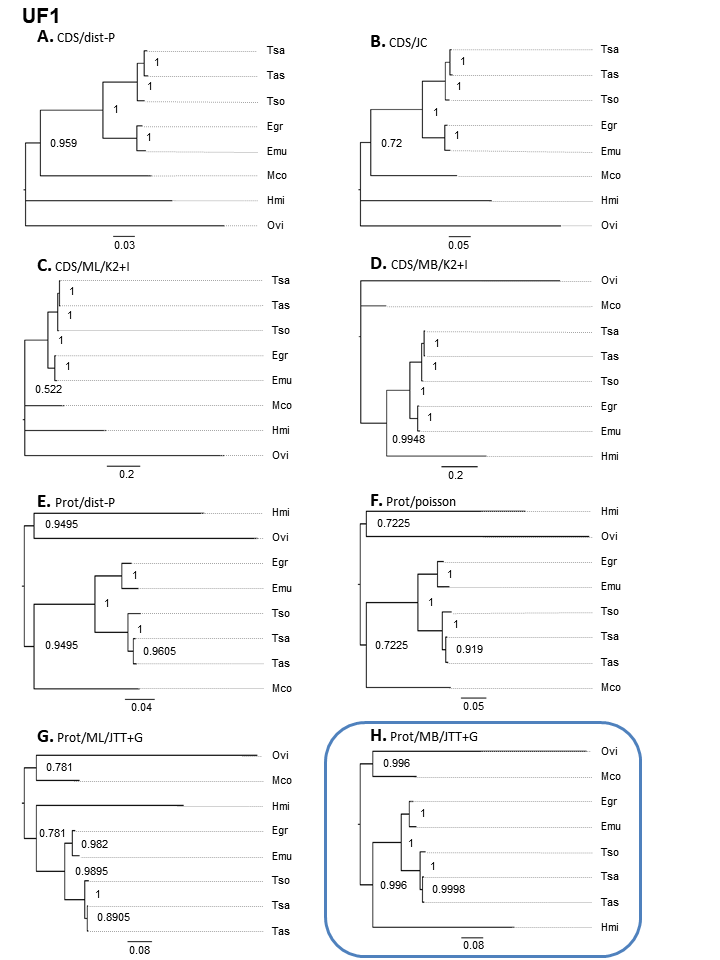
**

**Fig S16. UF1 phylogenetic analysis.** (A-D) Phylogenetic trees based on CDS alignments; they were built using p-distance (A), Jukes-Cantor (B), maximum likelihood by K2 with gamma distribution (C), and bayesian by K2 with gamma distribution models (D). (E-H) Phylogenetic trees based on protein alignments; they were built using p-distance (E); Poisson (F), maximum likelihood by JTT with gamma distribution (G) and bayesian by JTT with gamma distribution models (H). The best phylogenetic tree is highlighted by a blue box. The analyzed species were Egr, *Echinococcus granulosus*; Emu, *Echinococcus multilocularis*; Hmi, *Hymenolepis microstoma*; Mco, *Mesocestoides corti*; Ovi, *Opisthorchis viverrini*; Tas, *Taenia asiatica*; Tsa, *Taenia saginata*; and Tso, *Taenia solium*. CDS and protein alignments are shown in Table S6.

**
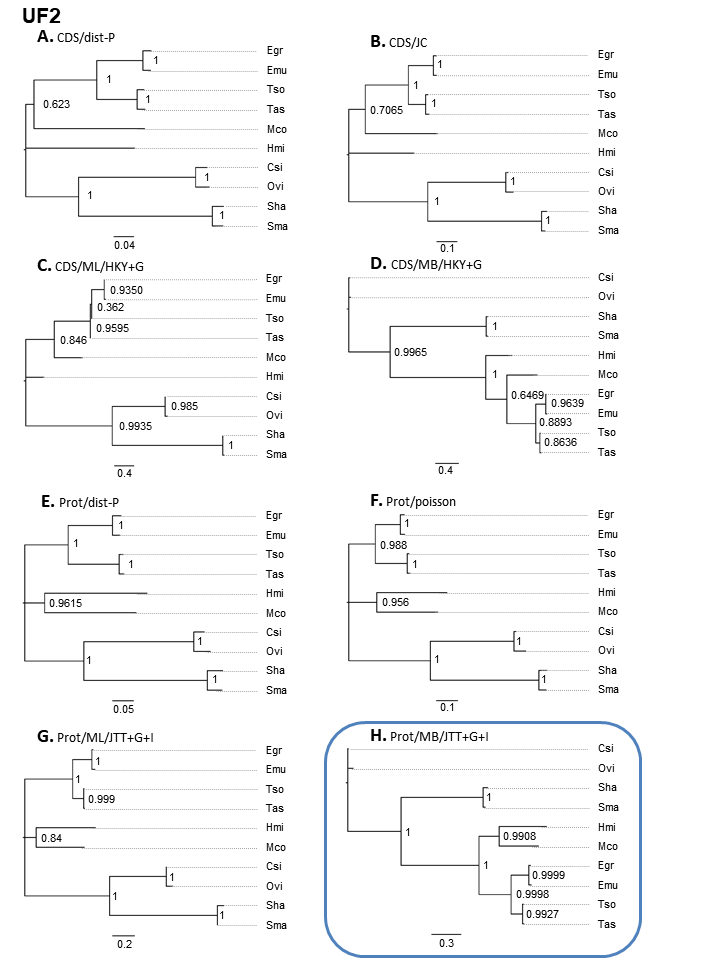
**

**Fig S17. UF2 phylogenetic analysis.** (A-D) Phylogenetic trees based on CDS alignments; they were built using p-distance (A), Jukes-Cantor (B), maximum likelihood by K2 with gamma distribution (C), and bayesian by K2 with gamma distribution models (D). (E-H) Phylogenetic trees based on protein alignments; they were built using p-distance (E); Poisson (F), maximum likelihood by JTT with gamma distribution (G) and bayesian by JTT with gamma distribution models (H). The best phylogenetic tree is highlighted by a blue box. The analyzed species were Csi, *Clonorchis sinensis*; Egr, *Echinococcus granulosus*; Emu, *Echinococcus multilocularis*; Hmi, *Hymenolepis microstoma*; Mco, *Mesocestoides corti*; Ovi, *Opisthorchis viverrini*; Sha, *Schistosoma haematobium*; Sma, *Schistosoma mansoni*; Tas, *Taenia asiatica*; and Tso, *Taenia solium*. CDS and protein alignments are shown in Table S6.

**
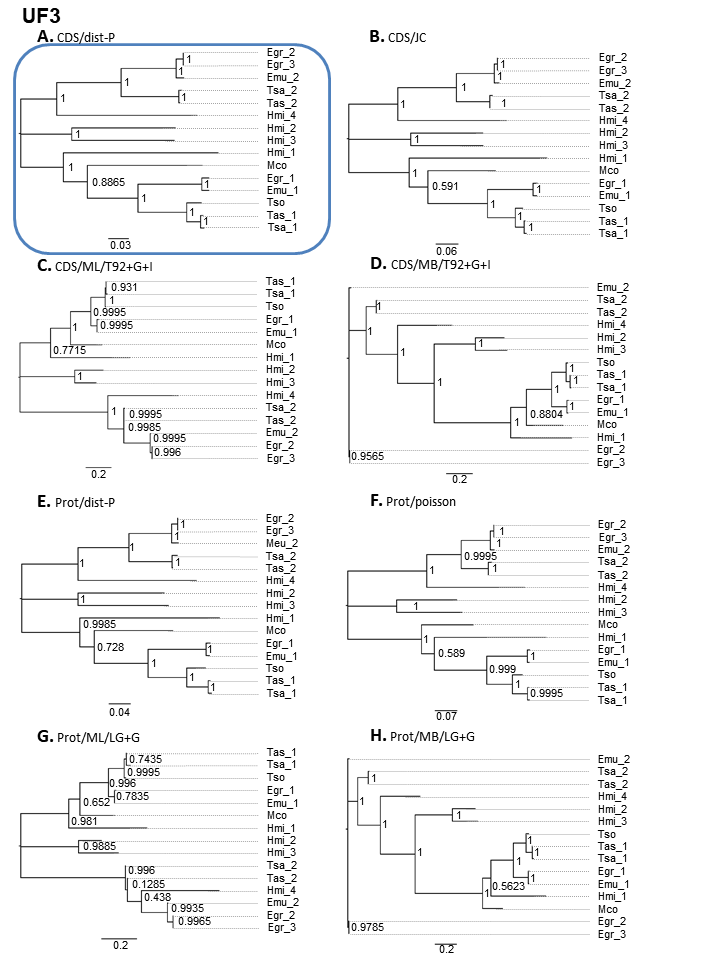
**

**Fig S18. UF3 phylogenetic analysis.** (A-D) Phylogenetic trees based on CDS alignments; they were built using p-distance (A), Jukes-Cantor (B), maximum likelihood by K2 with gamma distribution (C), and bayesian by K2 with gamma distribution models (D). (E-H) Phylogenetic trees based on protein alignments; they were built using p-distance (E); Poisson (F), maximum likelihood by JTT with gamma distribution (G) and bayesian by JTT with gamma distribution models (H). The best phylogenetic tree is highlighted by a blue box. The analyzed species were Egr, *Echinococcus granulosus*; Emu, *Echinococcus multilocularis*; Hmi, *Hymenolepis microstoma*; Mco, *Mesocestoides corti*; Tas, *Taenia asiatica*; Tsa, *Taenia saginata*; and Tso, *Taenia solium*. CDS and protein alignments are shown in Table S6.

**
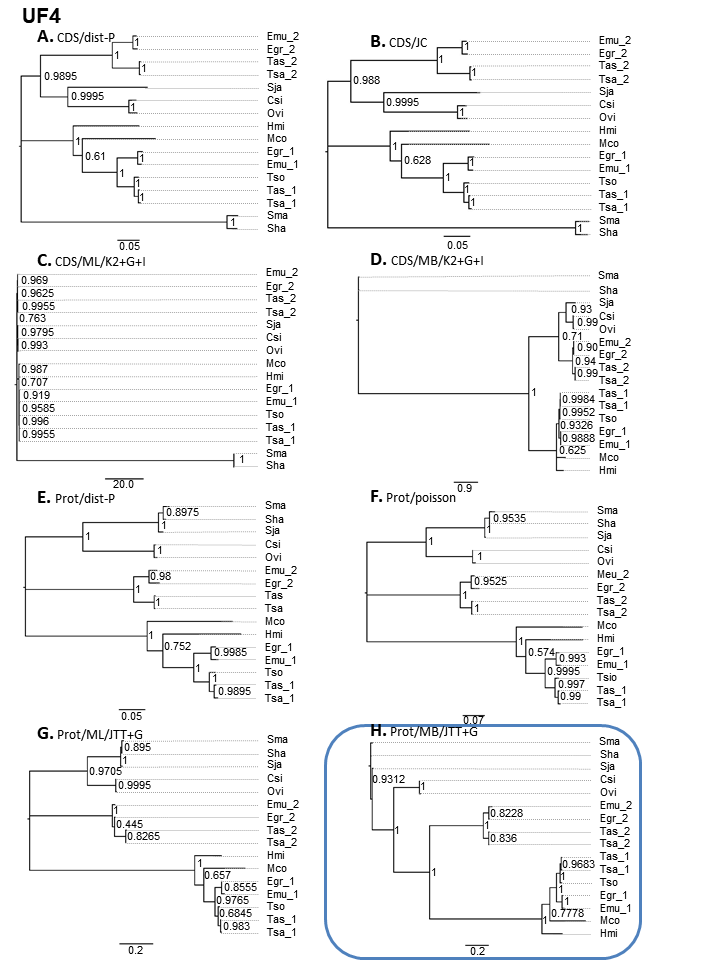
**

**Fig S19. UF4 phylogenetic analysis.** (A-D) Phylogenetic trees based on CDS alignments; they were built using p-distance (A), Jukes-Cantor (B), maximum likelihood by K2 with gamma distribution (C), and bayesian by K2 with gamma distribution models (D). (E-H) Phylogenetic trees based on protein alignments; they were built using p-distance (E); Poisson (F), maximum likelihood by JTT with gamma distribution (G) and bayesian by JTT with gamma distribution models (H). The best phylogenetic tree is highlighted by a blue box. The analyzed species were Csi, *Clonorchis sinensis*; Egr, *Echinococcus granulosus*; Emu, *Echinococcus multilocularis*; Hmi, *Hymenolepis microstoma*; Mco, *Mesocestoides corti*; Ovi, *Opisthorchis viverrini*; Sha, *Schistosoma haematobium*; Sja, *Schistosoma japonicum*; Sma, *Schistosoma mansoni*; Tas, *Taenia asiatica*; Tsa, *Taenia saginata*; and Tso, *Taenia solium*. CDS and protein alignments are shown in Table S6.

**
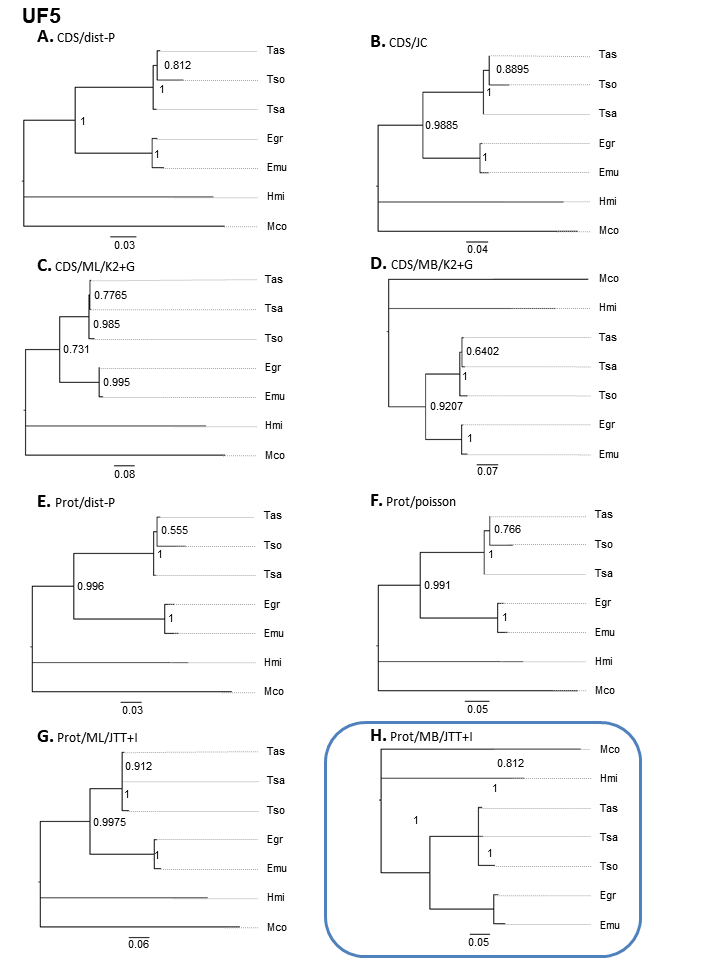
**

**Fig S20. UF5 phylogenetic analysis.** (A-D) Phylogenetic trees based on CDS alignments; they were built using p-distance (A), Jukes-Cantor (B), maximum likelihood by K2 with gamma distribution (C), and bayesian by K2 with gamma distribution models (D). (E-H) Phylogenetic trees based on protein alignments; they were built using p-distance (E); Poisson (F), maximum likelihood by JTT with gamma distribution (G) and bayesian by JTT with gamma distribution models (H). The best phylogenetic tree is highlighted by a blue box. The analyzed species were Egr, *Echinococcus granulosus*; Emu, *Echinococcus multilocularis*; Hmi, *Hymenolepis microstoma*; Mco, *Mesocestoides corti*; Tas, *Taenia asiatica*; Tsa, *Taenia saginata*; and Tso, *Taenia solium*. CDS and protein alignments are shown in Table S6.

**
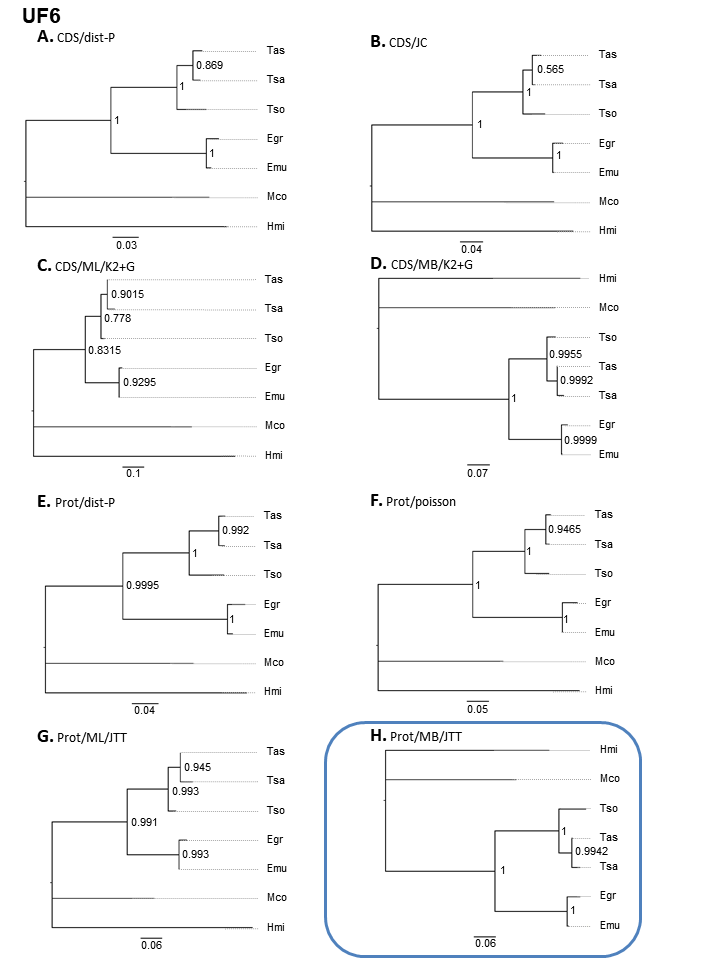
**

**Fig S21. UF6 phylogenetic analysis.** (A-D) Phylogenetic trees based on CDS alignments; they were built using p-distance (A), Jukes-Cantor (B), maximum likelihood by K2 with gamma distribution (C), and bayesian by K2 with gamma distribution models (D). (E-H) Phylogenetic trees based on protein alignments; they were built using p-distance (E); Poisson (F), maximum likelihood by JTT with gamma distribution (G) and bayesian by JTT with gamma distribution models (H). The best phylogenetic tree is highlighted by a blue box. The analyzed species were Egr, *Echinococcus granulosus*; Emu, *Echinococcus multilocularis*; Hmi, *Hymenolepis microstoma*; Mco, *Mesocestoides corti*; Tas, *Taenia asiatica*; Tsa, *Taenia saginata*; and Tso, *Taenia solium*. CDS and protein alignments are shown in Table S6.

**
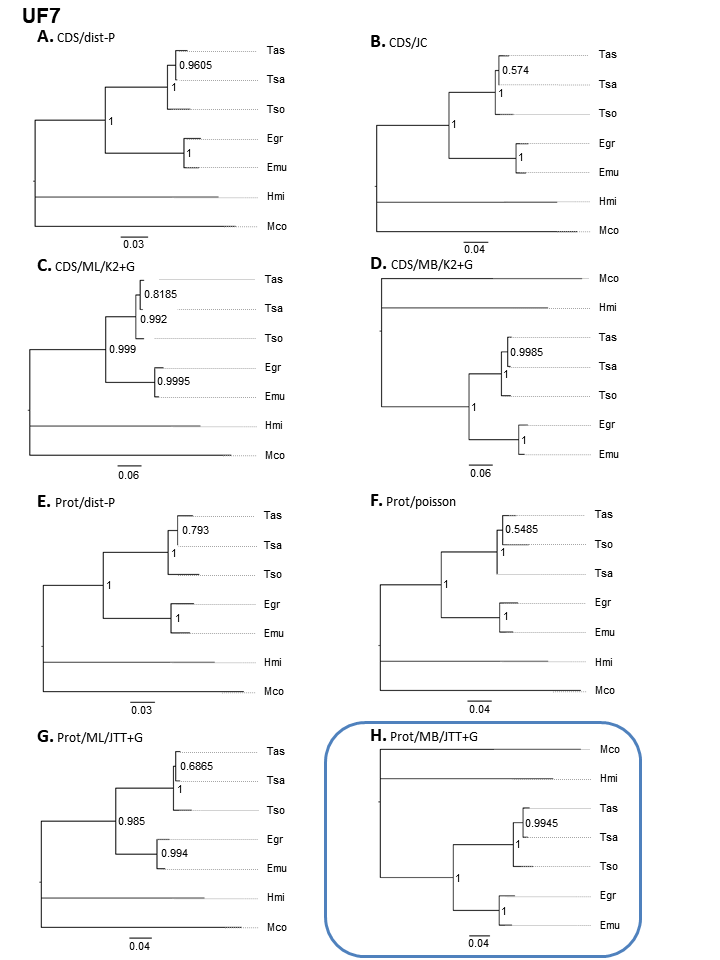
**

**Fig S22. UF7 phylogenetic analysis.** (A-D) Phylogenetic trees based on CDS alignments; they were built using p-distance (A), Jukes-Cantor (B), maximum likelihood by K2 with gamma distribution (C), and bayesian by K2 with gamma distribution models (D). (E-H) Phylogenetic trees based on protein alignments; they were built using p-distance (E); Poisson (F), maximum likelihood by JTT with gamma distribution (G) and bayesian by JTT with gamma distribution models (H). The best phylogenetic tree is highlighted by a blue box. The analyzed species were Egr, *Echinococcus granulosus*; Emu, *Echinococcus multilocularis*; Hmi, *Hymenolepis microstoma*; Mco, *Mesocestoides corti*; Tas, *Taenia asiatica*; Tsa, *Taenia saginata*; and Tso, *Taenia solium*. CDS and protein alignments are shown in Table S6.

**
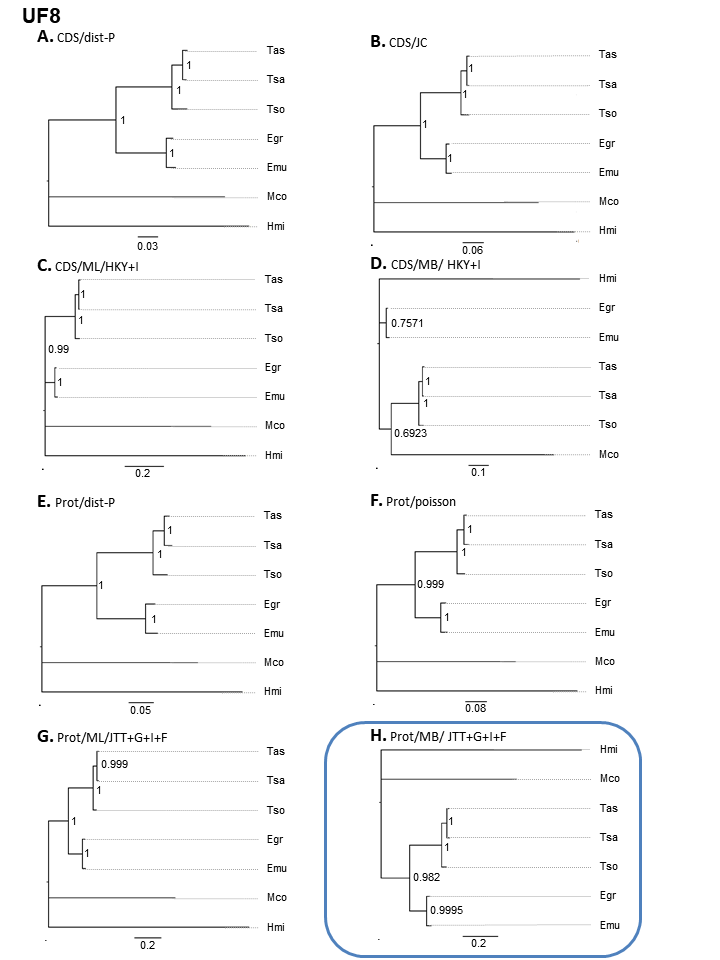
**

**Fig S23. UF8 phylogenetic analysis.** (A-D) Phylogenetic trees based on CDS alignments; they were built using p-distance (A), Jukes-Cantor (B), maximum likelihood by K2 with gamma distribution (C), and bayesian by K2 with gamma distribution models (D). (E-H) Phylogenetic trees based on protein alignments; they were built using p-distance (E); Poisson (F), maximum likelihood by JTT with gamma distribution (G) and bayesian by JTT with gamma distribution models (H). The best phylogenetic tree is highlighted by a blue box. The analyzed species were Egr, *Echinococcus granulosus*; Emu, *Echinococcus multilocularis*; Hmi, *Hymenolepis microstoma*; Mco, *Mesocestoides corti*; Tas, *Taenia asiatica*; Tsa, *Taenia saginata*; and Tso, *Taenia solium*. CDS and protein alignments are shown in Table S6.

**
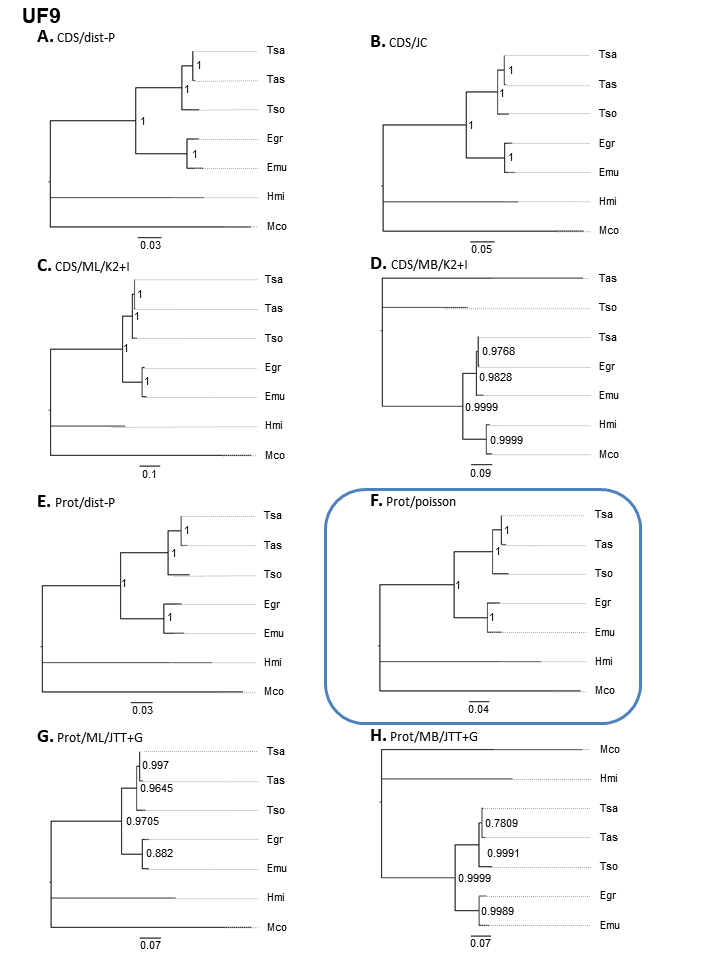
**

**Fig S24. UF9 phylogenetic analysis.** (A-D) Phylogenetic trees based on CDS alignments; they were built using p-distance (A), Jukes-Cantor (B), maximum likelihood by K2 with gamma distribution (C), and bayesian by K2 with gamma distribution models (D). (E-H) Phylogenetic trees based on protein alignments; they were built using p-distance (E); Poisson (F), maximum likelihood by JTT with gamma distribution (G) and bayesian by JTT with gamma distribution models (H). The best phylogenetic tree is highlighted by a blue box. The analyzed species were Egr, *Echinococcus granulosus*; Emu, *Echinococcus multilocularis*; Hmi, *Hymenolepis microstoma*; Mco, *Mesocestoides corti*; Tas, *Taenia asiatica*; Tsa, *Taenia saginata*; and Tso, *Taenia solium*. CDS and protein alignments are shown in Table S6.

**
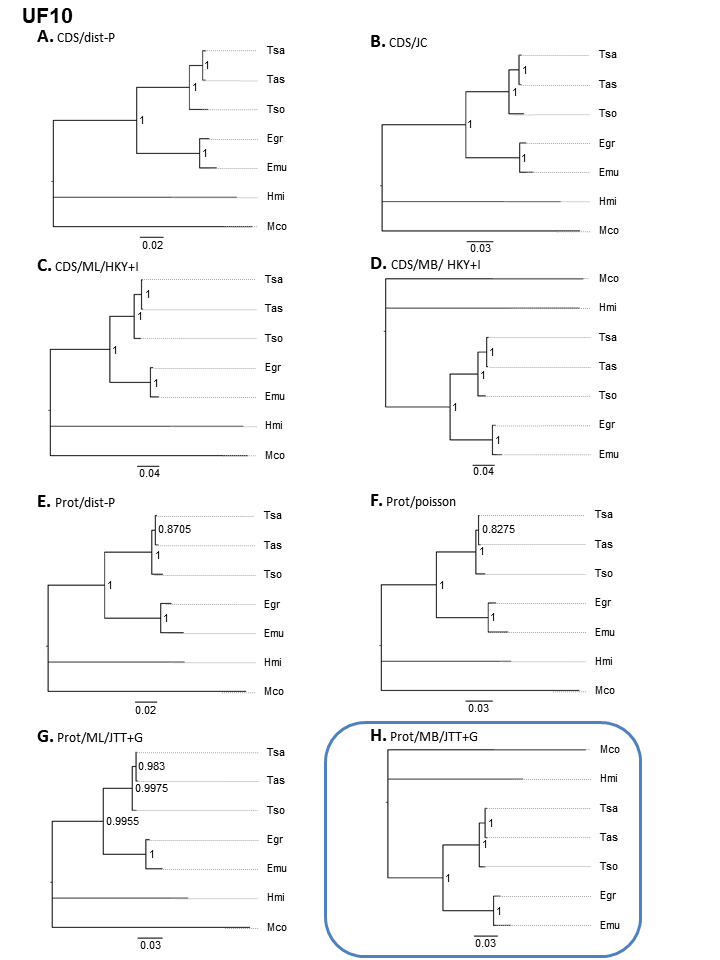
**

**Fig S25. UF10 phylogenetic analysis.** (A-D) Phylogenetic trees based on CDS alignments; they were built using p-distance (A), Jukes-Cantor (B), maximum likelihood by K2 with gamma distribution (C), and bayesian by K2 with gamma distribution models (D). (E-H) Phylogenetic trees based on protein alignments; they were built using p-distance (E); Poisson (F), maximum likelihood by JTT with gamma distribution (G) and bayesian by JTT with gamma distribution models (H). The best phylogenetic tree is highlighted by a blue box. The analyzed species were Egr, *Echinococcus granulosus*; Emu, *Echinococcus multilocularis*; Hmi, *Hymenolepis microstoma*; Mco, *Mesocestoides corti*; Tas, *Taenia asiatica*; Tsa, *Taenia saginata*; and Tso, *Taenia solium*. CDS and protein alignments are shown in Table S6.

**
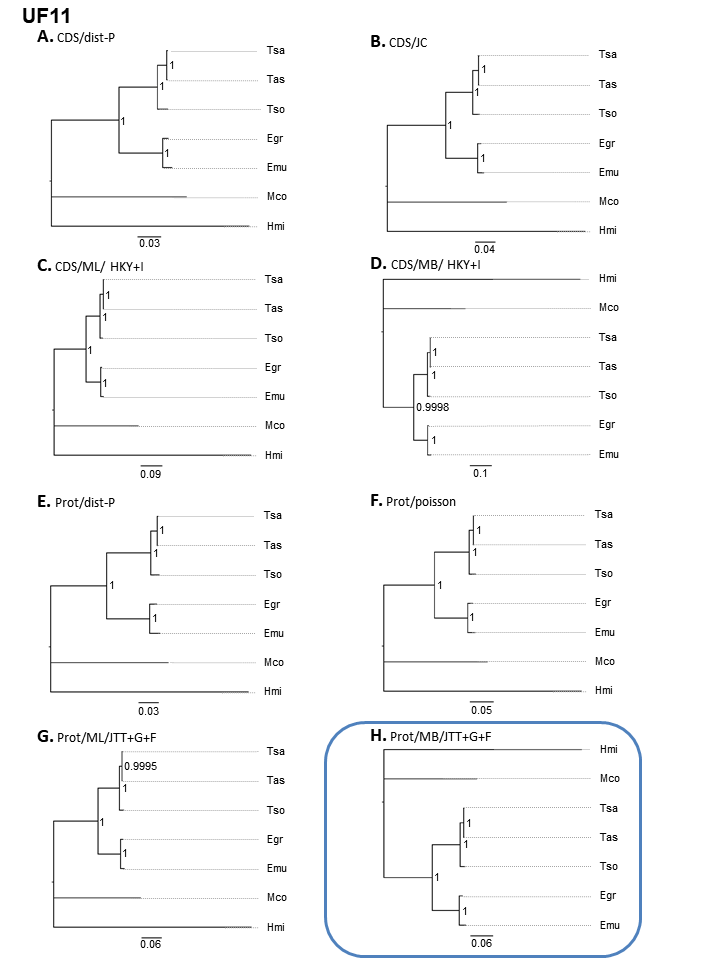
**

**Fig S26. UF11 phylogenetic analysis.** (A-D) Phylogenetic trees based on CDS alignments; they were built using p-distance (A), Jukes-Cantor (B), maximum likelihood by K2 with gamma distribution (C), and bayesian by K2 with gamma distribution models (D). (E-H) Phylogenetic trees based on protein alignments; they were built using p-distance (E); Poisson (F), maximum likelihood by JTT with gamma distribution (G) and bayesian by JTT with gamma distribution models (H). The best phylogenetic tree is highlighted by a blue box. The analyzed species were Egr, *Echinococcus granulosus*; Emu, *Echinococcus multilocularis*; Hmi, *Hymenolepis microstoma*; Mco, *Mesocestoides corti*; Tas, *Taenia asiatica*; Tsa, *Taenia saginata*; and Tso, *Taenia solium*. CDS and protein alignments are shown in Table S6.

**
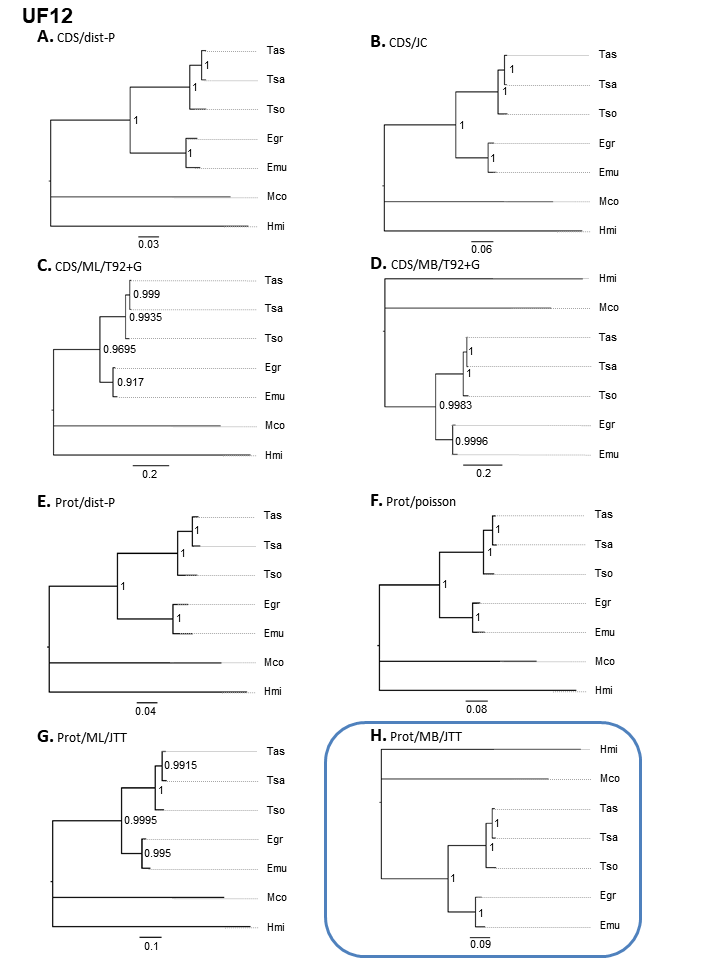
**

**Fig S27. UF12 phylogenetic analysis.** (A-D) Phylogenetic trees based on CDS alignments; they were built using p-distance (A), Jukes-Cantor (B), maximum likelihood by K2 with gamma distribution (C), and bayesian by K2 with gamma distribution models (D). (E-H) Phylogenetic trees based on protein alignments; they were built using p-distance (E); Poisson (F), maximum likelihood by JTT with gamma distribution (G) and bayesian by JTT with gamma distribution models (H). The best phylogenetic tree is highlighted by a blue box. The analyzed species were Egr, *Echinococcus granulosus*; Emu, *Echinococcus multilocularis*; Hmi, *Hymenolepis microstoma*; Mco, *Mesocestoides corti*; Tas, *Taenia asiatica*; Tsa, *Taenia saginata*; and Tso, *Taenia solium*. CDS and protein alignments are shown in Table S6.

**
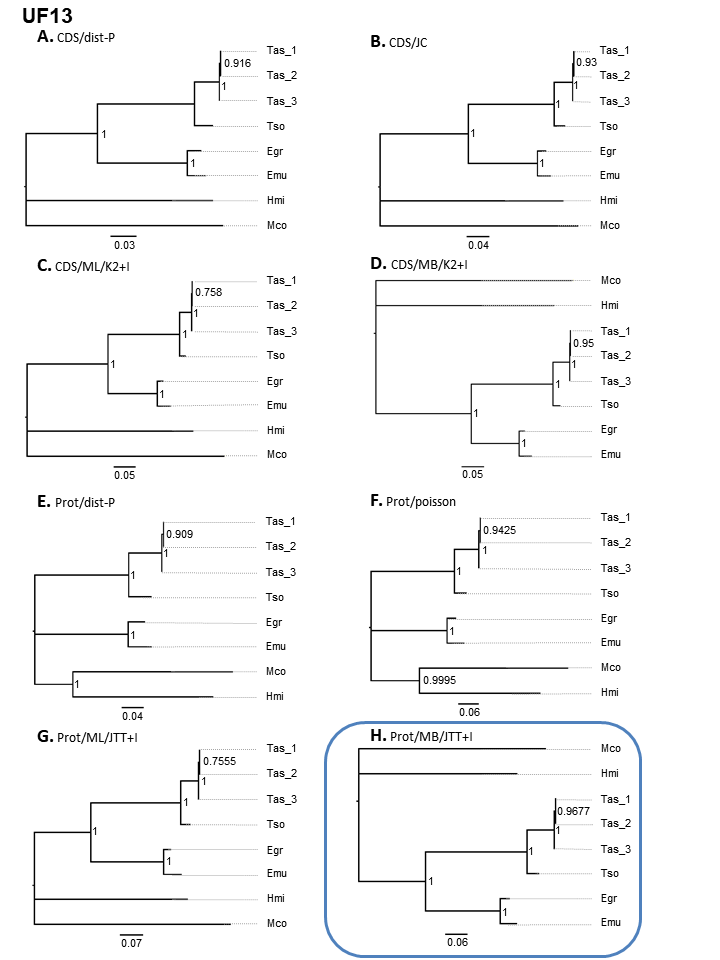
**

**Fig S28. UF13 phylogenetic analysis.** (A-D) Phylogenetic trees based on CDS alignments; they were built using p-distance (A), Jukes-Cantor (B), maximum likelihood by K2 with gamma distribution (C), and bayesian by K2 with gamma distribution models (D). (E-H) Phylogenetic trees based on protein alignments; they were built using p-distance (E); Poisson (F), maximum likelihood by JTT with gamma distribution (G) and bayesian by JTT with gamma distribution models (H). The best phylogenetic tree is highlighted by a blue box. The analyzed species were Egr, *Echinococcus granulosus*; Emu, *Echinococcus multilocularis*; Hmi, *Hymenolepis microstoma*; Mco, *Mesocestoides corti*; Tas, *Taenia asiatica*; and Tso, *Taenia solium*. CDS and protein alignments are shown in Table S6.

**
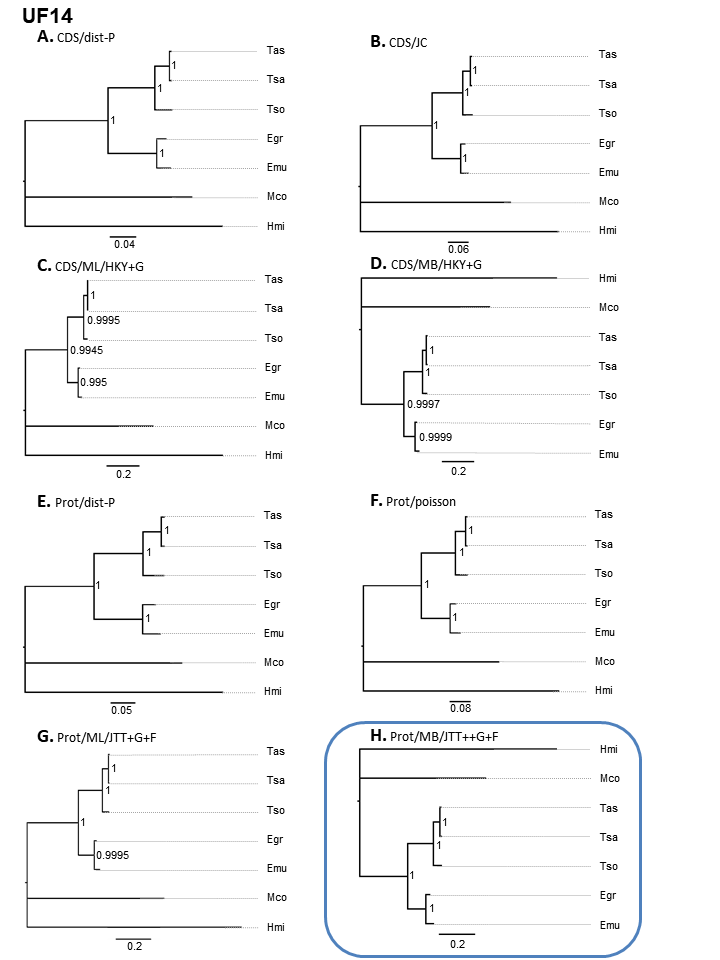
**

**Fig S29. UF14 phylogenetic analysis.** (A-D) Phylogenetic trees based on CDS alignments; they were built using p-distance (A), Jukes-Cantor (B), maximum likelihood by K2 with gamma distribution (C), and bayesian by K2 with gamma distribution models (D). (E-H) Phylogenetic trees based on protein alignments; they were built using p-distance (E); Poisson (F), maximum likelihood by JTT with gamma distribution (G) and bayesian by JTT with gamma distribution models (H). The best phylogenetic tree is highlighted by a blue box. The analyzed species were Egr, *Echinococcus granulosus*; Emu, *Echinococcus multilocularis*; Hmi, *Hymenolepis microstoma*; Mco, *Mesocestoides corti*; Tas, *Taenia asiatica*; Tsa, *Taenia saginata*; and Tso, *Taenia solium*. CDS and protein alignments are shown in Table S6.

**
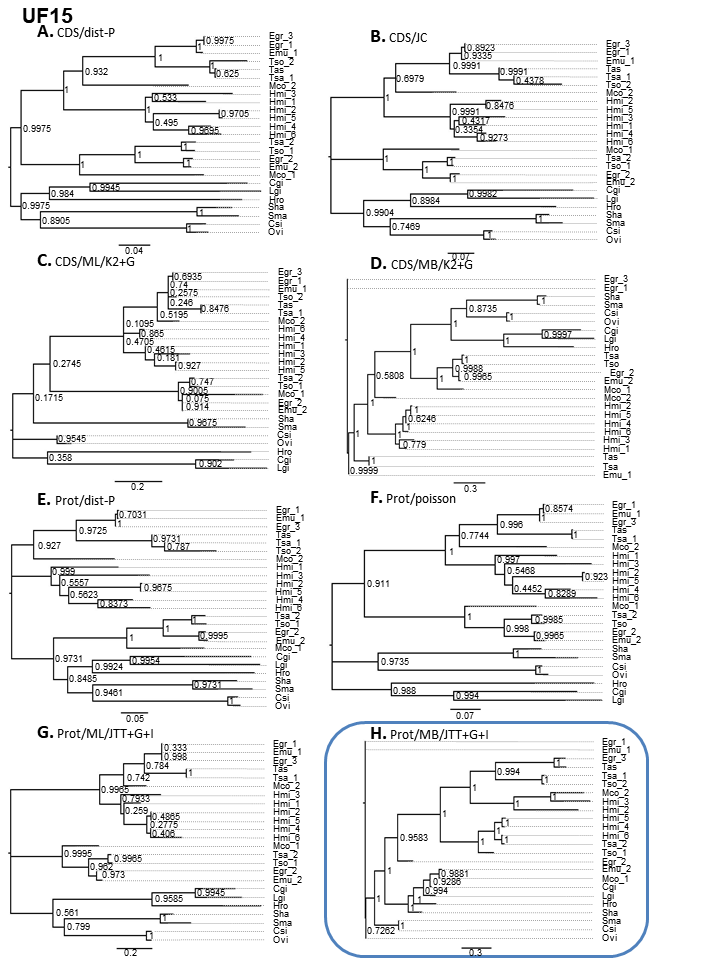
**

**Fig S30. UF15 phylogenetic analysis.** (A-D) Phylogenetic trees based on CDS alignments; they were built using p-distance (A), Jukes-Cantor (B), maximum likelihood by K2 with gamma distribution (C), and bayesian by K2 with gamma distribution models (D). (E-H) Phylogenetic trees based on protein alignments; they were built using p-distance (E); Poisson (F), maximum likelihood by JTT with gamma distribution (G) and bayesian by JTT with gamma distribution models (H). The best phylogenetic tree is highlighted by a blue box. The analyzed species were Cgi, *Crassostrea gigas*; Csi, *Clonorchis sinensis*; Egr, *Echinococcus granulosus*; Emu, *Echinococcus multilocularis*; Hmi, *Hymenolepis microstoma*; Hro, *Helobdella robusta*; Lgi, *Lottia gigantea*; Mco, *Mesocestoides corti*; Ovi, *Opisthorchis viverrini*; Sha, *Schistosoma haematobium*; Sma, *Schistosoma mansoni*; Tas, *Taenia asiatica*; Tsa, *Taenia saginata*; and Tso, *Taenia solium*. CDS and protein alignments are shown in Table S6.

**
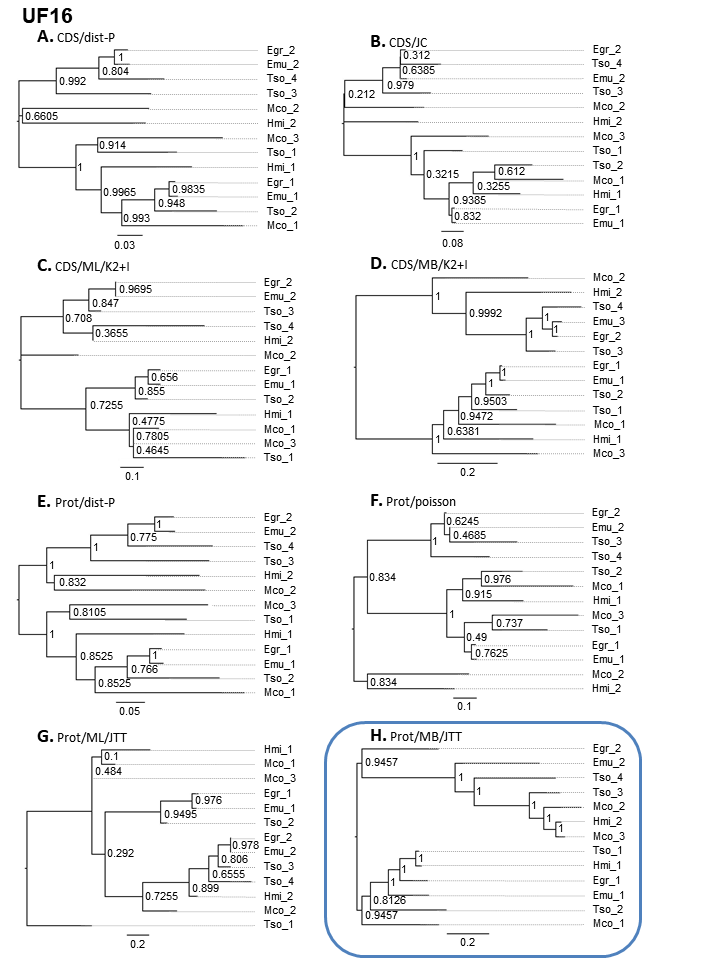
**

**Fig S31. UF16 phylogenetic analysis.** (A-D) Phylogenetic trees based on CDS alignments; they were built using p-distance (A), Jukes-Cantor (B), maximum likelihood by K2 with gamma distribution (C), and bayesian by K2 with gamma distribution models (D). (E-H) Phylogenetic trees based on protein alignments; they were built using p-distance (E); Poisson (F), maximum likelihood by JTT with gamma distribution (G) and bayesian by JTT with gamma distribution models (H). The best phylogenetic tree is highlighted by a blue box. The analyzed species were Egr, *Echinococcus granulosus*; Emu, *Echinococcus multilocularis*; Hmi, *Hymenolepis microstoma*; Mco, *Mesocestoides corti*; and Tso, *Taenia solium*. CDS and protein alignments are shown in Table S6.

**
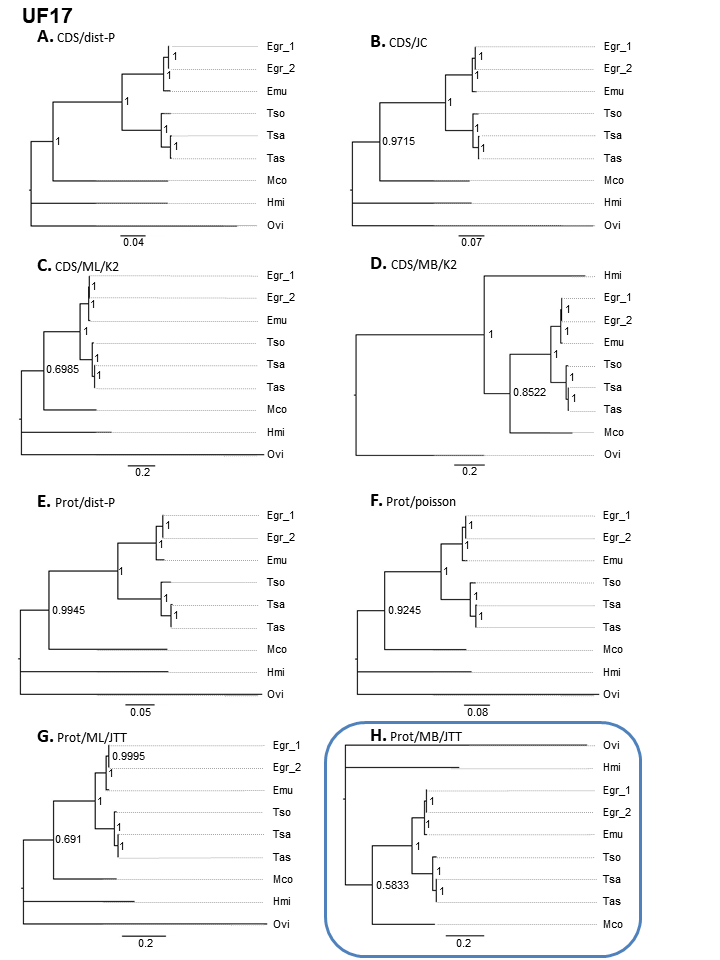
**

**Fig S32. UF17 phylogenetic analysis.** (A-D) Phylogenetic trees based on CDS alignments; they were built using p-distance (A), Jukes-Cantor (B), maximum likelihood by K2 with gamma distribution (C), and bayesian by K2 with gamma distribution models (D). (E-H) Phylogenetic trees based on protein alignments; they were built using p-distance (E); Poisson (F), maximum likelihood by JTT with gamma distribution (G) and bayesian by JTT with gamma distribution models (H). The best phylogenetic tree is highlighted by a blue box. The analyzed species were Egr, *Echinococcus granulosus*; Emu, *Echinococcus multilocularis*; Hmi, *Hymenolepis microstoma*; Mco, *Mesocestoides corti*; Tas, *Taenia asiatica*; Ovi, *Opisthorchis viverrini*; Tsa, *Taenia saginata*; and Tso, *Taenia solium*. CDS and protein alignments are shown in Table S6.

**
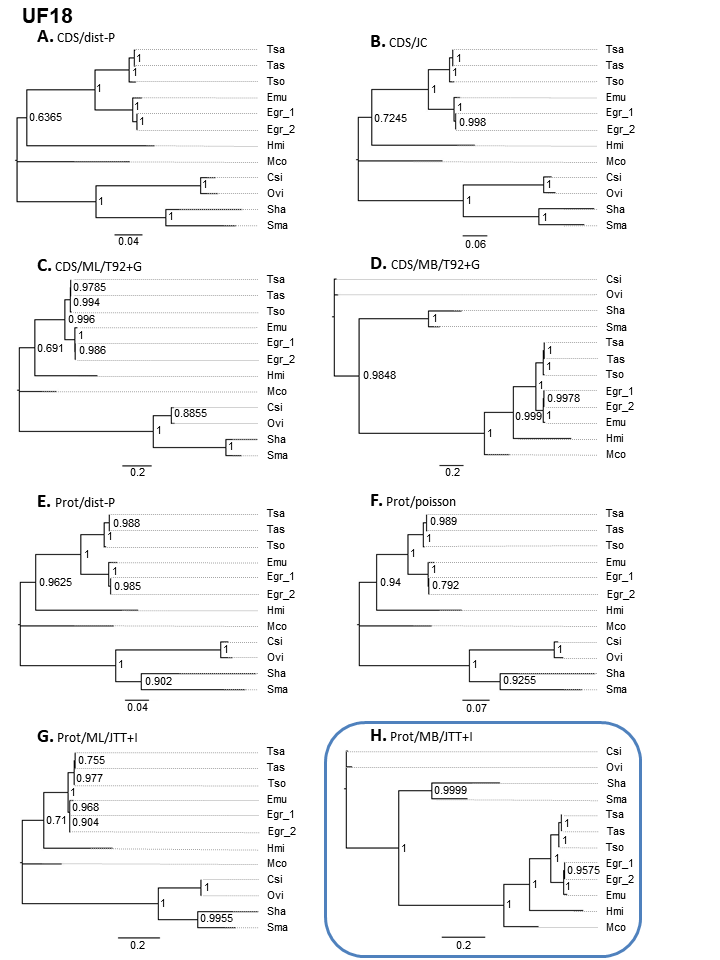
**

**Fig S33. UF18 phylogenetic analysis.** (A-D) Phylogenetic trees based on CDS alignments; they were built using p-distance (A), Jukes-Cantor (B), maximum likelihood by K2 with gamma distribution (C), and bayesian by K2 with gamma distribution models (D). (E-H) Phylogenetic trees based on protein alignments; they were built using p-distance (E); Poisson (F), maximum likelihood by JTT with gamma distribution (G) and bayesian by JTT with gamma distribution models (H). The best phylogenetic tree is highlighted by a blue box. The analyzed species were Csi, *Clonorchis sinensis*; Egr, *Echinococcus granulosus*; Emu, *Echinococcus multilocularis*; Hmi, *Hymenolepis microstoma*; Mco, *Mesocestoides corti*; Ovi, *Opisthorchis viverrini*; Sha, *Schistosoma haematobium*; Sma, *Schistosoma mansoni*; Tas, *Taenia asiatica*; Tsa, *Taenia saginata*; and Tso, *Taenia solium*. CDS and protein alignments are shown in Table S6.

**
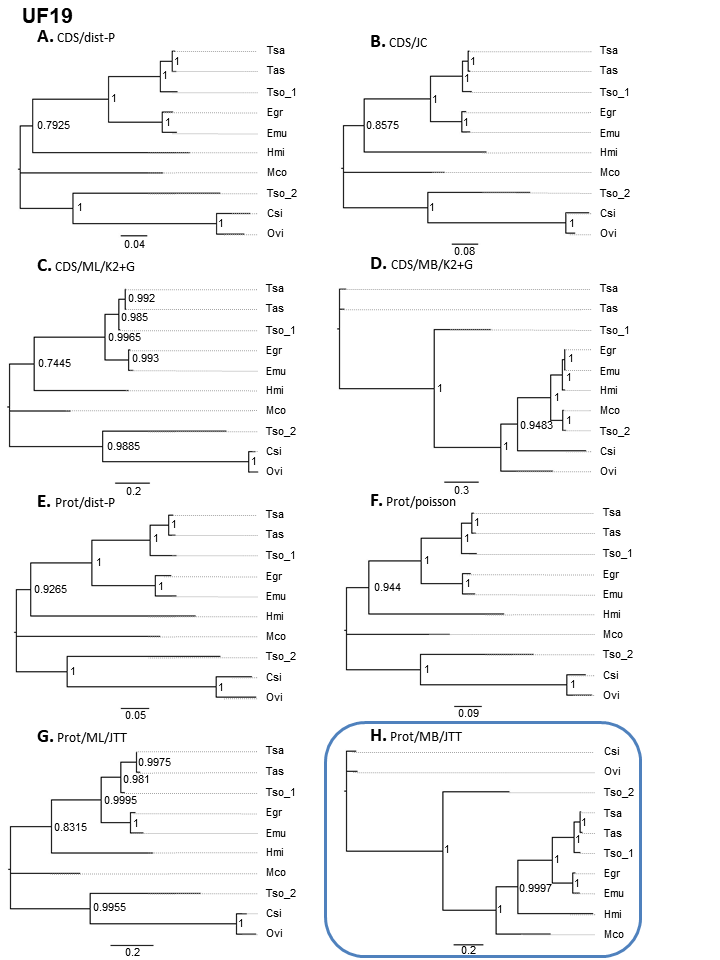
**

**Fig S34. UF19 phylogenetic analysis.** (A-D) Phylogenetic trees based on CDS alignments; they were built using p-distance (A), Jukes-Cantor (B), maximum likelihood by K2 with gamma distribution (C), and bayesian by K2 with gamma distribution models (D). (E-H) Phylogenetic trees based on protein alignments; they were built using p-distance (E); Poisson (F), maximum likelihood by JTT with gamma distribution (G) and bayesian by JTT with gamma distribution models (H). The best phylogenetic tree is highlighted by a blue box. The analyzed species were Csi, *Clonorchis sinensis*; Egr, *Echinococcus granulosus*; Emu, *Echinococcus multilocularis*; Hmi, *Hymenolepis microstoma*; Mco, *Mesocestoides corti*; Ovi, *Opisthorchis viverrini*; Tas, *Taenia asiatica*; Tsa, *Taenia saginata*; and Tso, *Taenia solium*. CDS and protein alignments are shown in Table S6.

**
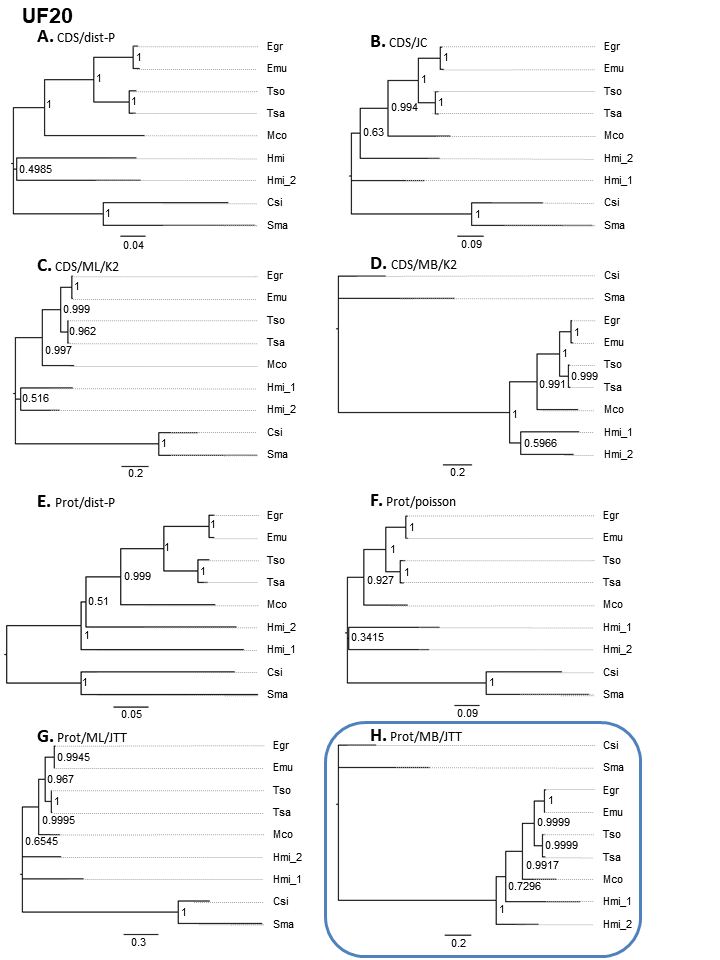
**

**Fig S35. UF20 phylogenetic analysis.** (A-D) Phylogenetic trees based on CDS alignments; they were built using p-distance (A), Jukes-Cantor (B), maximum likelihood by K2 with gamma distribution (C), and bayesian by K2 with gamma distribution models (D). (E-H) Phylogenetic trees based on protein alignments; they were built using p-distance (E); Poisson (F), maximum likelihood by JTT with gamma distribution (G) and bayesian by JTT with gamma distribution models (H). The best phylogenetic tree is highlighted by a blue box. The analyzed species were Csi, *Clonorchis sinensis*; Egr, *Echinococcus granulosus*; Emu, *Echinococcus multilocularis*; Hmi, *Hymenolepis microstoma*; Mco, *Mesocestoides corti*; Sma, *Schistosoma mansoni*; Tsa, *Taenia saginata*; and Tso, *Taenia solium*. CDS and protein alignments are shown in Table S6.

**
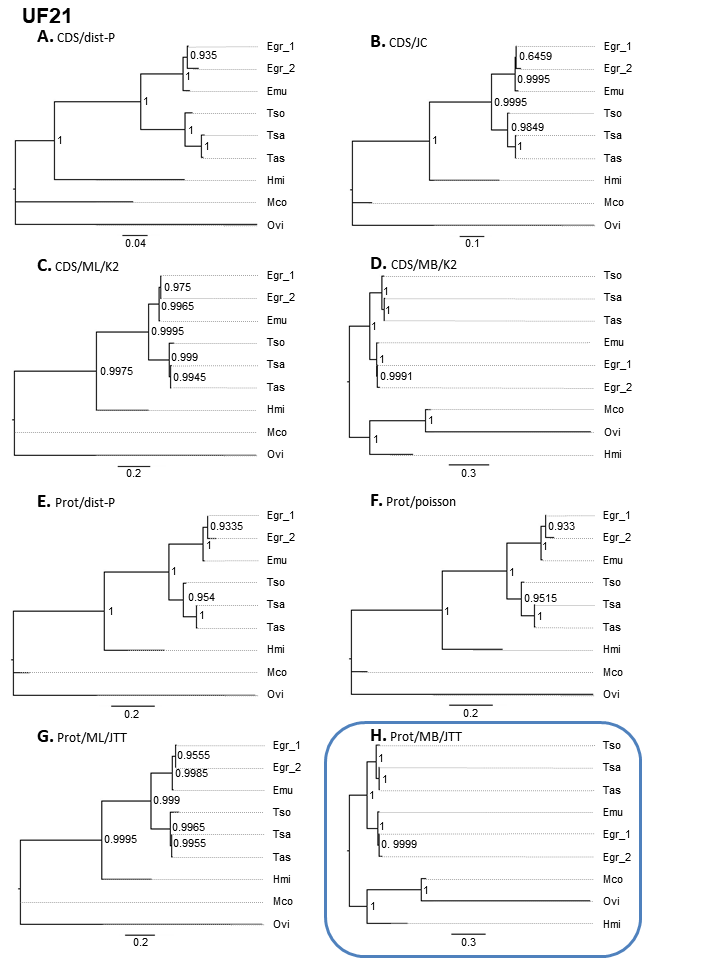
**

**Fig S36. UF21 phylogenetic analysis.** (A-D) Phylogenetic trees based on CDS alignments; they were built using p-distance (A), Jukes-Cantor (B), maximum likelihood by K2 with gamma distribution (C), and bayesian by K2 with gamma distribution models (D). (E-H) Phylogenetic trees based on protein alignments; they were built using p-distance (E); Poisson (F), maximum likelihood by JTT with gamma distribution (G) and bayesian by JTT with gamma distribution models (H). The best phylogenetic tree is highlighted by a blue box. The analyzed species were Egr, *Echinococcus granulosus*; Emu, *Echinococcus multilocularis*; Hmi, *Hymenolepis microstoma*; Mco, *Mesocestoides corti*; Ovi, *Opisthorchis viverrini*; Tas, *Taenia asiatica*; Tsa, *Taenia saginata*; and Tso, *Taenia solium*. CDS and protein alignments are shown in Table S6.

**
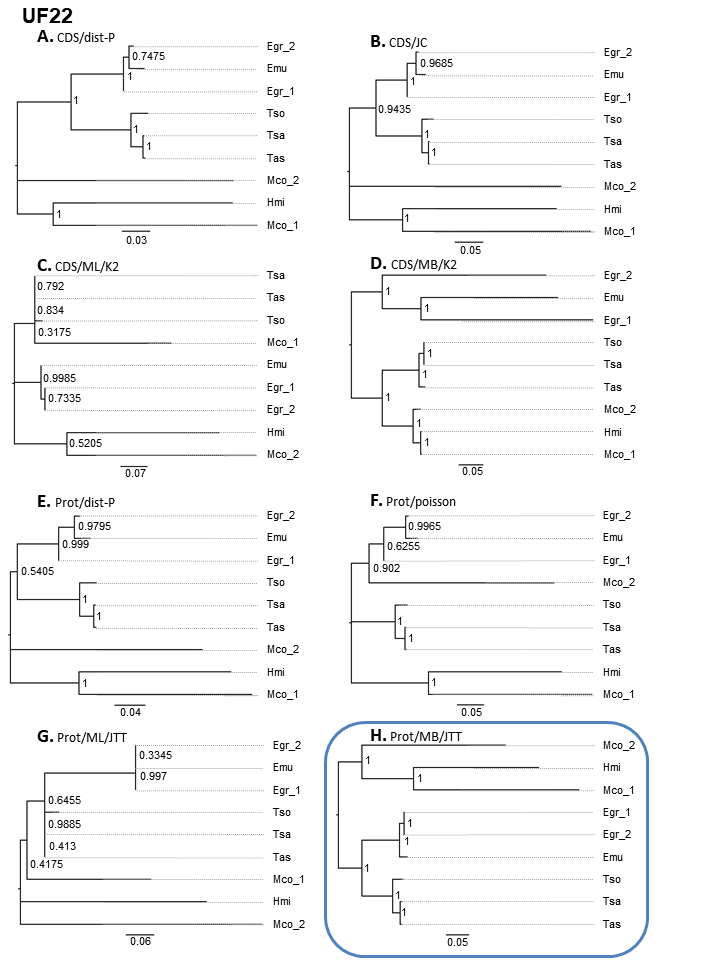
**

**Fig S37. UF22 phylogenetic analysis.** (A-D) Phylogenetic trees based on CDS alignments; they were built using p-distance (A), Jukes-Cantor (B), maximum likelihood by K2 with gamma distribution (C), and bayesian by K2 with gamma distribution models (D). (E-H) Phylogenetic trees based on protein alignments; they were built using p-distance (E); Poisson (F), maximum likelihood by JTT with gamma distribution (G) and bayesian by JTT with gamma distribution models (H). The best phylogenetic tree is highlighted by a blue box. The analyzed species were Egr, *Echinococcus granulosus*; Emu, *Echinococcus multilocularis*; Hmi, *Hymenolepis microstoma*; Mco, *Mesocestoides corti*; Tas, *Taenia asiatica*; Tsa, *Taenia saginata*; and Tso, *Taenia solium*. CDS and protein alignments are shown in Table S6.

**
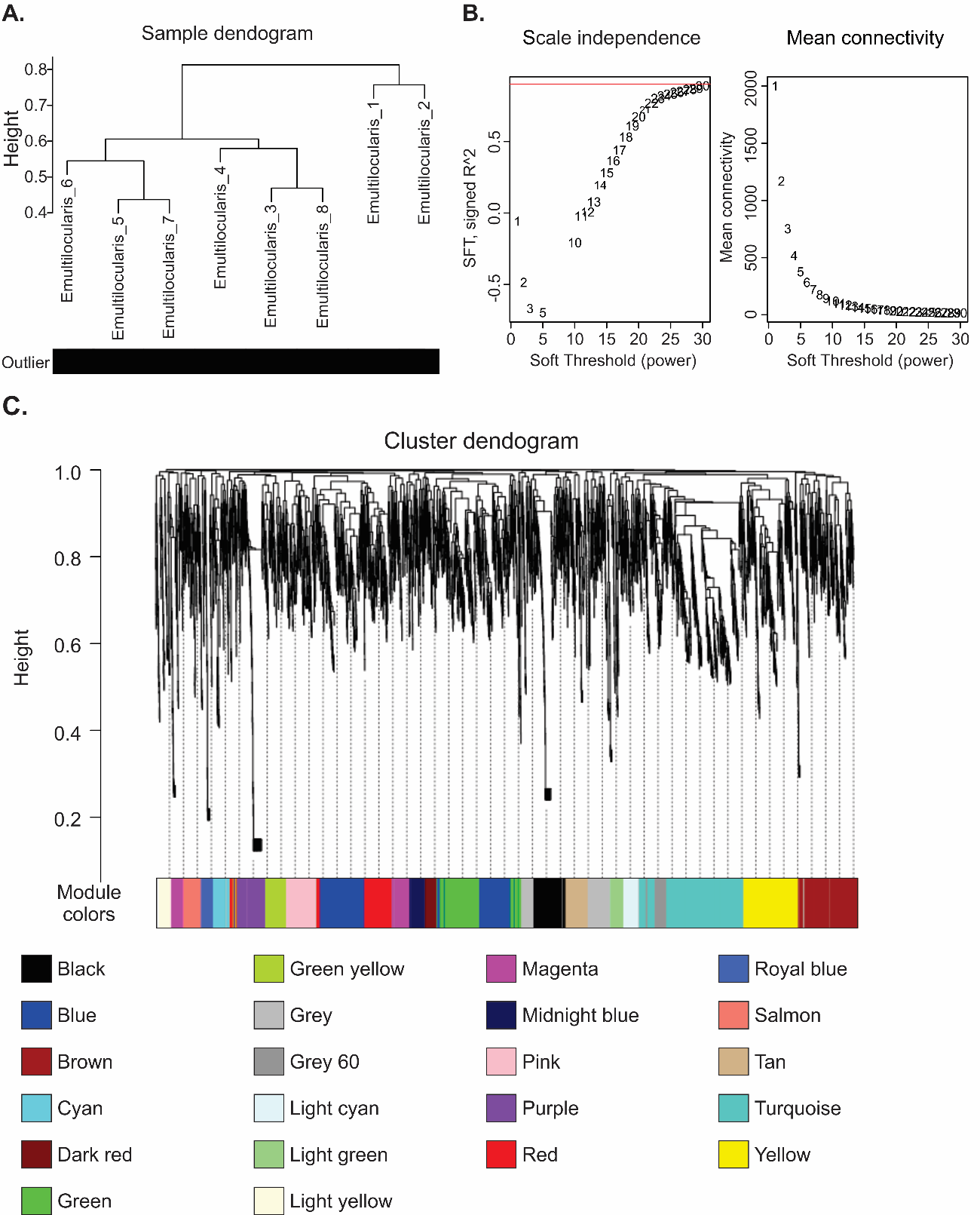
**

**Fig S38. Gene co-expression analysis.** (A) Relationships among RNA-seq samples; no outliers were identified. (B) Determination of the parameter β of the adjacency function in the weighted gene correlation network analysis (WGCNA) algorithm. (C) Hierarchical cluster tree showings the co-expression modules identified by WGCNA; modules were color-named as indicated.

**
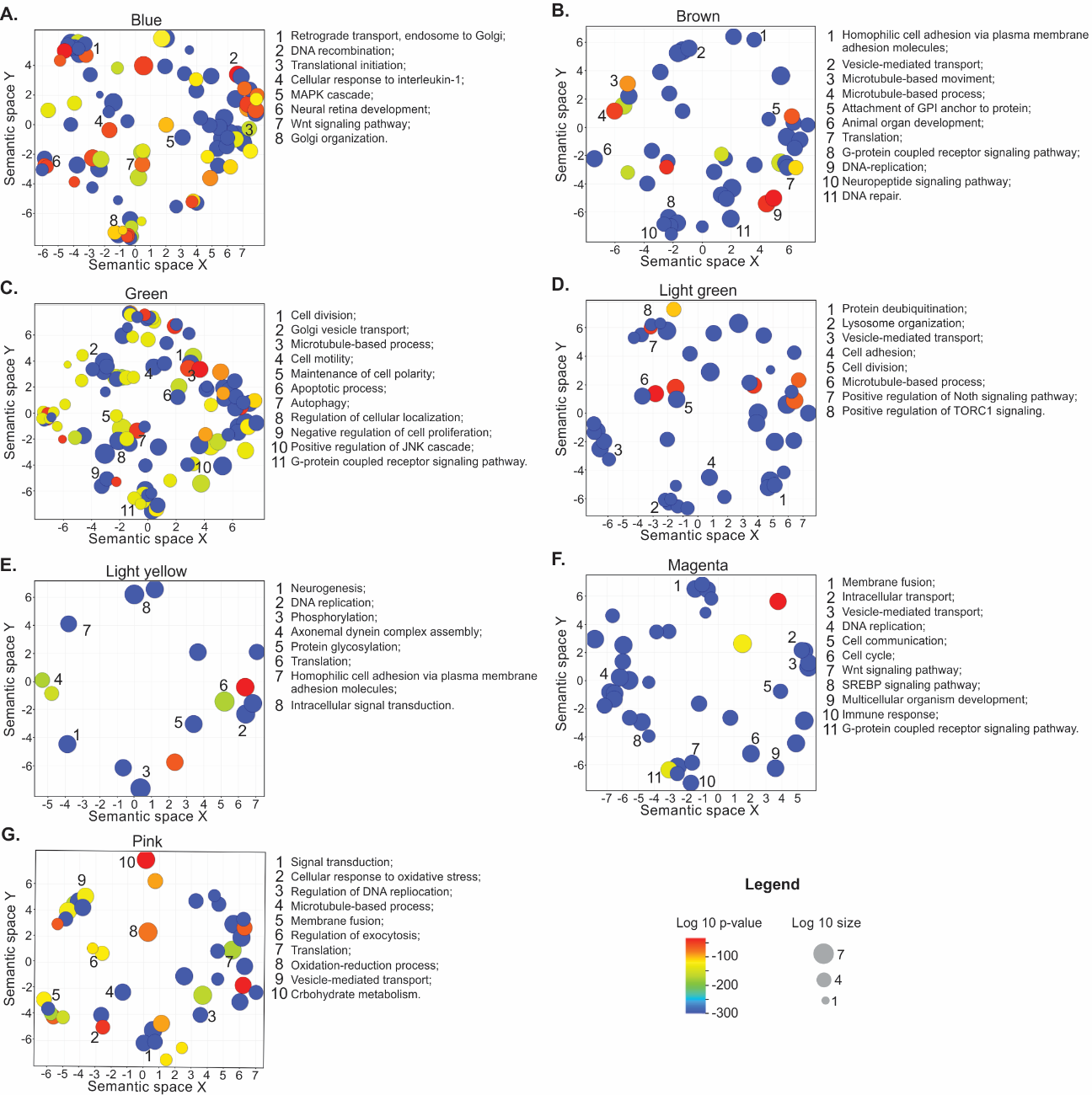
**

**Fig S39. Module functional enrichment.** The scatterplots show the cluster terms remaining after the redundancy reduction in a two dimensional space generated by Revigo. Bubble colors indicate the p-values and their sizes indicate the GO term frequencies. Results are shows for the following modules (A) blue, (B) brown, (C) green, (D) light green, (E) light yellow, (F) magenta and (G) pink.
